# In the Absence of Sonic Hedgehog, p53 Induces Apoptosis and Inhibits Retinal Cell Proliferation, Cell-Cycle Exit and Differentiation in Zebrafish

**DOI:** 10.1371/journal.pone.0013549

**Published:** 2010-10-21

**Authors:** Sergey V. Prykhozhij

**Affiliations:** Developmental Biology Unit, European Molecular Biology Laboratory, Heidelberg, Germany; University of Dayton, United States of America

## Abstract

**Background:**

Sonic hedgehog (Shh) signaling regulates cell proliferation during vertebrate development via induction of cell-cycle regulator gene expression or activation of other signalling pathways, prevents cell death by an as yet unclear mechanism and is required for differentiation of retinal cell types. Thus, an unsolved question is how the same signalling molecule can regulate such distinct cell processes as proliferation, cell survival and differentiation.

**Methodology/Principal Findings:**

Analysis of the zebrafish *shh* ^−/−^ mutant revealed that in this context p53 mediates elevated apoptosis during nervous system and retina development and interferes with retinal proliferation and differentiation. While in *shh* ^−/−^ mutants there is activation of p53 target genes and p53-mediated apoptosis, an increase in Hedgehog (Hh) signalling by over-expression of dominant-negative Protein Kinase A strongly decreased p53 target gene expression and apoptosis levels in *shh* ^−/−^ mutants. Using a novel p53 reporter transgene, I confirm that p53 is active in tissues that require Shh for cell survival. Proliferation assays revealed that loss of *p53* can rescue normal cell-cycle exit and the mitotic indices in the *shh* ^−/−^ mutant retina at 24, 36 and 48 hpf. Moreover, generation of amacrine cells and photoreceptors was strongly enhanced in the double *p53* ^−/−^ *shh* ^−/−^ mutant retina suggesting the effect of p53 on retinal differentiation.

**Conclusions:**

Loss of Shh signalling leads to the p53-dependent apoptosis in the developing nervous system and retina. Moreover, Shh-mediated control of p53 activity is required for proliferation and cell cycle exit of retinal cells as well as differentiation of amacrine cells and photoreceptors.

## Introduction

During organogenesis, cell numbers are tightly controlled by balancing proliferation and cell death. Hh signaling pathway regulates cell proliferation and survival during development in addition to its pattern formation and differentiation functions in many different biological contexts (reviewed in [1], [2], [3]). Work from many labs has shown that secreted Hh proteins bind to the transmembrane protein Patched (Ptc) and release the inhibition of the transmembrane protein Smoothened (Smo) by Ptc. The signal transduction downstream of Smo inhibits proteolytic processing of Gli2 and Gli3 to repressive forms, which is mediated by Protein Kinase A (PKA) and a few other factors [4]. The resulting full-length Gli factors can activate their target genes.

Shh is an essential survival factor for many cell types during development such as neural stem cells [5], cells in the neural tube [6], [7], [8], midbrain and forebrain [9], neural crest [10], [11], retina [12], and ventral sclerotome [7]. These findings are supported by the fact that restoring Shh signalling can suppress apoptosis [6], [8]. Interestingly, in the neural tube Shh signalling promotes cell survival before its requirement for tissue patterning and differentiation [13] arguing against the hypothesis that Shh prevents cell death by providing patterning cues.

Nevertheless, there is still some controversy about the underlying mechanism of the anti-apoptotic role of Shh. Activation of anti-apoptotic genes by Shh signaling is the simplest scenario, and indeed, Gli1 can activate BCL2 gene expression in human cell lines and in the mouse keratinocytes by binding to several near-consensus sites in the BCL2 promoter [14]. Likewise, over-expression of the Gli3 dominant-active form in developing chick embryos leads to induction of *Bcl2* gene [13]. Moreover, another study has proposed that in the absence of Shh, Patched1 mediates apoptosis in the neural tube through caspase activation independent of the canonical Shh signaling pathway [15]. Patched1 could function in this context as a dependency receptor, i.e. a receptor inducing cell death in the absence of cognate ligand binding [16], [17]. Recently, Abe and colleagues (2008) have shown that activation of Hh signaling can suppress p53 pathway in human cell lines [18]. The authors have proposed that Hh signaling induces an unknown Mdm2- activating factor leading to phosphorylation of serines 166 and 186 on Hdm2 (human Mdm2), which becomes activated and catalyses ubiquitination of p53. Degradation of ubiquitinated p53 disrupts p53-mediated tumour suppression under conditions of DNA damage and oncogenic stress. Furthermore, Stecca and Ruiz i Altaba (2009) have described a negative feedback loop between p53 and Gli1 in transgenic mouse models [19]. The authors found that p53 inhibits activity of Gli1 by interfering with its normal nuclear localization and directing it to proteosomal degradation. Closing the loop, Gli1 overexpression leads to a striking increase of Mdm2 expression and down-regulation of p53 in the mouse brain and several cell lines.

Beside this anti-apoptotic function, Shh is also known to stimulate proliferation by direct regulation of cell-cycle promoting genes or through activation of secondary signaling pathways.

In particular, Hh ligands stimulate proliferation in the mouse skin [20], in the vertebrate central nervous system [13], [21], [22], [23], [24], [25], and in *Drosophila* wing [26], [27] and eye imaginal discs [28]. In the vertebrate retina, Shh stimulates proliferation of retinal progenitors *in vitro* [29], [30] and in the ciliary marginal zone [31]. Moreover, during mouse retina development Shh-mediated activation of *cyclinD1* expression is essential for proliferation and cell-cycle exit of retinal ganglion cell progenitors [32]. Consistent with this, in the zebrafish *shh* ^−/−^ mutant retina, proliferation is reduced during neurogenesis [12]. In *Xenopus*, Shh signalling has been shown to promote retinal progenitor proliferation by activating expression of cell cycle regulators, which shortens G1- and G2-phases of the cell cycle, and thereby drives cells towards the final mitosis and cell-cycle exit [33]. Interestingly, in several other contexts Hh signalling has been shown to negatively regulate cell cycle progression [34]. Indeed, in zebrafish *shh* ^−/−^ mutant retina and after Hh signaling inhibition by forskolin, most retinal cells fail to express *p57Kip2* cyclin-dependent kinase (CDK) inhibitor and do not exit the cell cycle [35], [36].

This study shows that loss of *shh* in zebrafish leads to p53-mediated apoptosis in the developing nervous system and retina. Consistent with this, activation of Hh signalling suppresses p53 activity and apoptosis in the *shh* ^−/−^ mutant. To monitor p53 activation, I created a p53 reporter transgenic line expressing nuclear green fluorescent protein under control of p53-sensitive promoter, verified by p53 knock-down and being induced by roscovitine treatment. By using this line, I have determined the spatio-temporal activation of p53 and apoptosis in the *shh* ^−/−^ mutant. Further, comparisons of double *p53* ^−/−^ *shh* ^−/−^ mutants with wild-type, and *shh* ^−/−^ mutant embryos identified p53-mediated inhibition of retinal progenitor proliferation, cell-cycle exit and differentiation of amacrine cells and photoreceptors. Taken together, these data provide novel genetic evidence that Shh regulates cell survival during nervous system and retina development as well as retinal proliferation and differentiation through inhibition of p53 pathway.

## Results

### p53 mediates apoptosis in *shh* ^−/−^ mutant via the intrinsic apoptotic pathway

**Figure 1:**
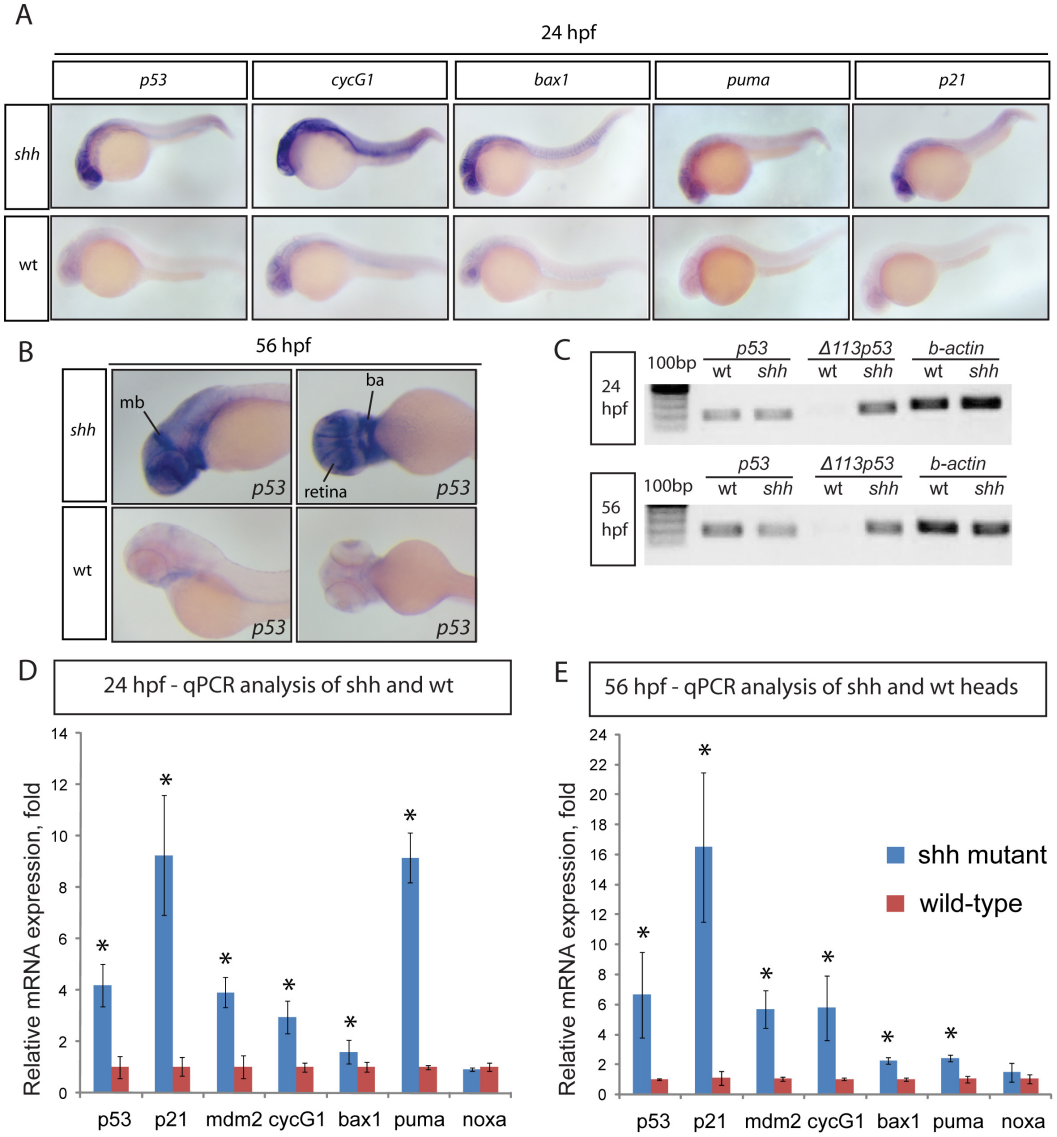
Induction of p53 target gene expression in the *shh* ^−/−^ mutant. (A) 24 hpf wild-type and *shh* ^−/−^ mutant embryos were *in situ* hybridised with probes against *p53, cyclinG1, bax1, puma* and *p21* mRNAs. These p53 target genes are expressed at a higher level in the *shh* ^−/−^ mutant in neural tube, retina and somites than in wild-type embryos. (B) 56 hpf wild-type and *shh* ^−/−^ mutant embryos were hybridized with the probe against *p53* mRNA. The hybridizations show increased expression of *p53* in *shh* ^−/−^ mutant compared to wild-type embryos in the brain, retina and branchial arches. The following structures are labelled: retina, midbrain (mb) and branchial arches (ba). (C) *p53* transcript isoforms in *shh* ^−/−^ mutant and wild-type at 24 and 56 hpf were analysed by semi-quantitative PCR assays for full-length *p53*, *Δ113 p53* transcripts and *β-actin* transcript as a loading control. At both stages, full-length *p53* and *β-actin* transcripts were expressed at similar levels in wild-type and mutant samples, whereas *Δ113 p53* was strongly upregulated in *shh* ^−/−^ mutant samples. (D, E) qPCR assays for *p53, p21, mdm2, cyclinG1, bax1, puma, noxa* and *gapdh* were performed at 24 hpf (D) and 56 hpf (E) on wild-type and *shh* ^−/−^ mutant embryos. cDNA from whole embryos was used for 24 hpf samples and cDNA from embryo heads was used for 56 hpf samples. Gene expression was normalized using *gapdh* expression as reference. The panels show p53 target gene expression in wild-type and *shh* ^−/−^ mutant embryos (D, E). Error bars represent standard deviation and an asterisk indicates statistical significance. There are significant expression differences between wild-type and *shh* ^−/−^ mutant samples (t-test, * - p<0.05) for all genes except *noxa*. qPCR assays were performed in triplicates on three different clutches of embryos and standard deviations are shown.

**Figure 2:**
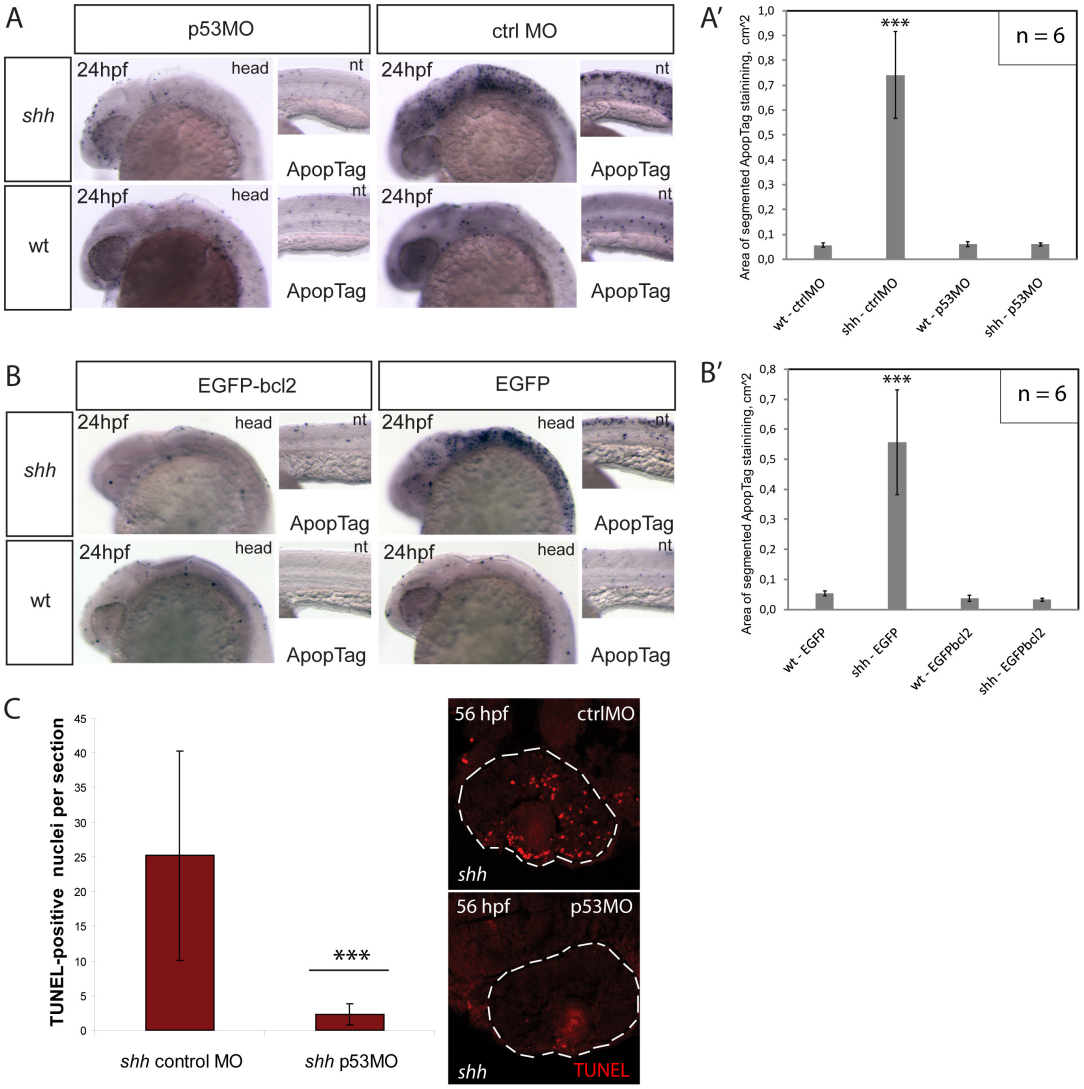
Knock-down of p53 suppresses apoptosis in the *shh* ^−/−^ mutant mediated by the intrinsic apoptosis pathway. (A) Apoptosis labelling using ApopTag staining in wild-type and *shh* ^−/−^ mutant embryos at 24 hpf after injection of p53 morpholino or control morpholino. The whole-mount images of the head (head) and neural tube (nt) are shown. (A') Quantification of ApopTag signal area in wild-type and *shh* ^−/−^ mutant embryo head images from (A) shows that p53MO successfully decreased apoptosis in *shh* ^−/−^ mutant to the wild-type level. Asterisks (***) on top of the *shh* ^−/−^ mutant bar indicate its significant statistical differences from other samples (t-test, P-value<0,001). (B) Apoptosis labelling using ApopTag staining in wild-type and *shh* ^−/−^ mutant embryos at 24 hpf after injection of of EGFP-bcl2 or EGFP mRNA. The whole-mount images of the head (head) and neural tube (nt) are shown. (B') Quantification of ApopTag signal area in wild-type and *shh* ^−/−^ mutant embryo head images from (B) shows that EGFP-bcl2 successfully decreased apoptosis in *shh* ^−/−^ mutant to the wild-type level. Asterisks (***) on top of the *shh* ^−/−^ mutant bar indicate its significant statistical differences from other samples (t-test, P-value<0,001). (C) Retinal apoptosis observed in *shh* ^−/−^ mutant at 56 hpf was suppressed by injection of p53 morpholino but not by injection of control morpholino (n = 10 and 40 sections for *shh* ^−/−^ mutant injected with p53 morpholino; n = 10 and 34 sections for *shh* ^−/−^ mutant injected with control morpholino). *** - t-test, P-value<0,001.

To determine the mechanism of cell death due to loss of *shh*, I analysed the expression of anti-apoptotic Bcl-2 family genes *bcl2*, *bcl2l*, *mcl1a* and *mcl1b* in *shh* ^−/−^ mutant and wild-type embryos, their expression being comparable in embryos of both genotypes at 24 hours post fertilization (hpf) (Fig. S1). I next looked at the expression of p53-dependent genes by *in situ* hybridisations which revealed consistently stronger expression of p53 target genes *p53*, *cyclinG1*, *bax1*, *puma* and *p21* at 24 hpf in the neural tube, retina and somites of *shh* ^−/−^ mutant relative to wild-type embryos (Fig. 1A) and elevated expression of *p53* at 56 hpf in the *shh* ^−/−^ mutant retina, midbrain and branchial arches (Fig. 1B). The observed elevated p53 expression was due to induction of the short Δ113 p53 transcript isoform [37], [38] in the *shh* ^−/−^ mutant, whereas the level of the full-length *p53* transcript was comparable between *shh* ^−/−^ mutant and wild-type embryos (Fig. 1C). To better measure p53 target gene expression levels, I performed quantitative polymerase chain reaction (qPCR) analyses on *shh* ^−/−^ mutant versus wild-type embryos at 24 hpf and 56 hpf confirming elevated expression of *p53*, *p21 mdm2*, *cycG1*, *bax1* and *puma* in *shh* ^−/−^ mutants at both stages (Fig. 1D, E). The pro-apoptotic genes *bax1* and *puma*, but not *noxa*, showed higher expression in *shh* ^−/−^ mutants compared to wild-type embryos at both 24 hpf (Fig. 1D) and 56 hpf (Fig. 1E), the relative expression of *puma*, the most potent inducer of mitochondrial apoptosis [39], [40], being higher in *shh* ^−/−^ mutant embryos at 24 hpf (Fig. 1D) than at 56 hpf (Fig. 1E) correlating with a higher level of apoptosis at 24 hpf (Fig. 2).

To confirm that p53 is essential for elevated apoptosis in the *shh* ^−/−^ mutant, I used morpholino oligonucleotides (MO) [41] knock-down against p53 previously shown to effectively block p53 translation during zebrafish development [42]. Injection of p53 MO but not of 4-mismatch control MO into the *shh* ^−/−^ mutant embryos suppressed elevated apoptosis in the neural tube at 24 hpf (Fig. 2A) as confirmed using image quantification of apoptosis stainings (Fig. 2A') and in the retina at 56 hpf (Fig. 2C). Apoptosis can occur through either extrinsic pathway when cells receive external signals activating death receptors or intrinsic (mitochondrial) pathway when pro-apoptotic factors are released from mitochondria after their permeabilization, p53 activating primarily the intrinsic rather than the extrinsic apoptotic pathway [43]. To test whether p53 activates the mitochondrial pathway of apoptosis in the *shh* ^−/−^ mutant, I injected mRNA of EGFP-bcl2, an inhibitor of the mitochondrial apoptotic pathway, or EGFP into *shh* ^−/−^ mutant and wild-type embryos. Expression of EGFP-bcl2 significantly reduced the level of apoptosis in *shh* ^−/−^ mutants at 24 hpf, whereas EGFP did not have any effect (Fig. 2B, B') suggesting that the p53-mediated apoptosis in the *shh* ^−/−^ mutant occurs via the intrinsic pathway.

### *p53*-dependent apoptosis in the *shh* ^−/−^ mutant retina

**Figure 3:**
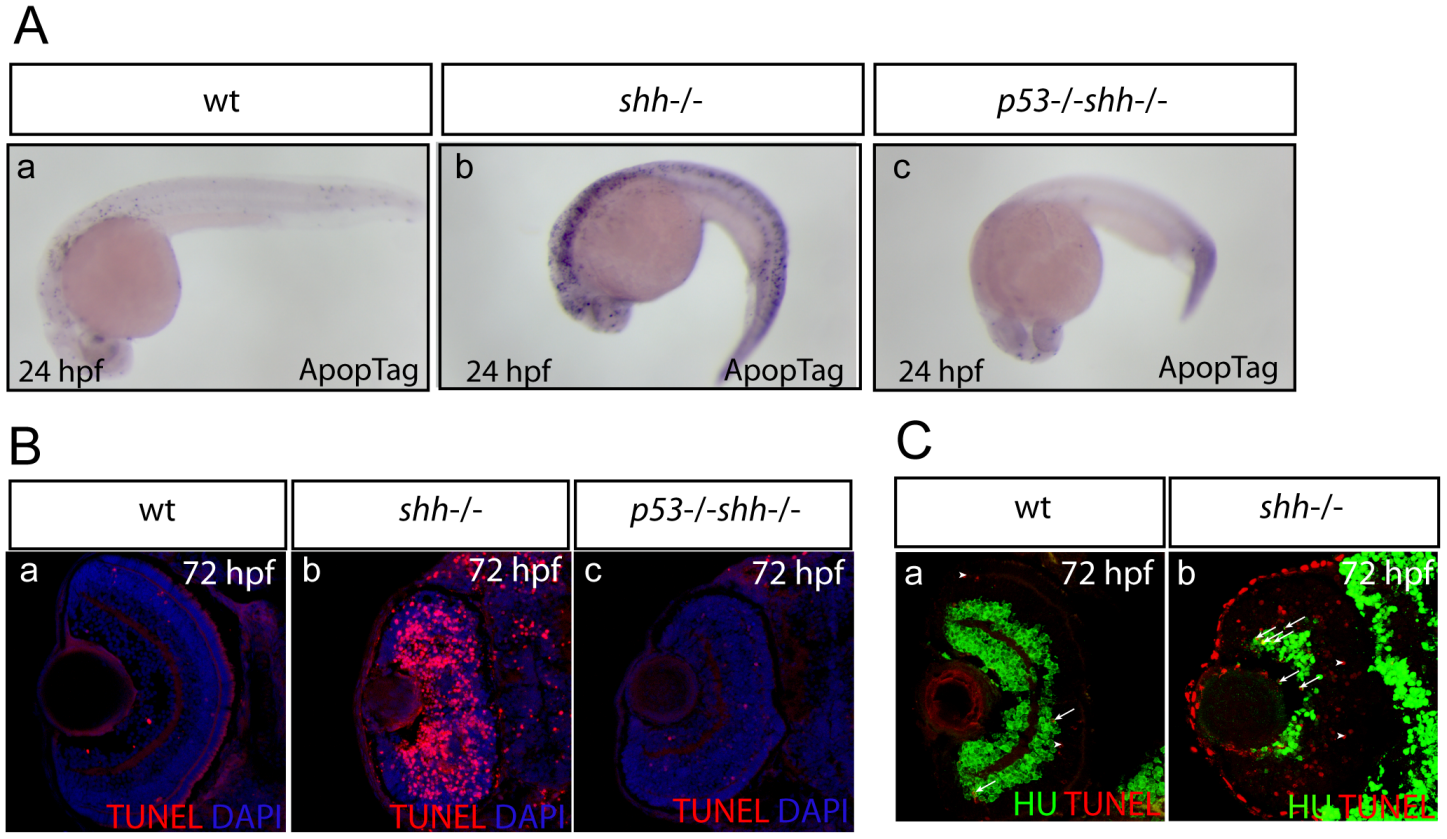
Genetic loss of *p53* suppresses apoptosis in the *shh* ^−/−^ mutant. Apoptosis was assayed in whole-mount embryos using ApopTag (A, a–c) and on retinal sections using TUNEL labelling (B, a–c). The sections are oriented with their anterior side to the top. Elevated apoptosis in *shh* ^−/−^ mutant embryos compared to wild-type embryos was suppressed by *p53* loss in the developing nervous system (A, a–c) and in the retina (B, a–c). (A) Whole-mount ApopTag staining at 24 hpf of wild-type, *shh* ^−/−^ and *p53* ^−/−^ *shh* ^−/−^ embryos (a–c). (B) TUNEL/DAPI staining of retinal sections of wild-type, *shh* ^−/−^ and *p53* ^−/−^ *shh* ^−/−^ embryos at 72 hpf (a–c). (C) Wild-type (a) and *shh* ^−/−^ mutant (b) retinal cryosections from 72 hpf embryos stained with anti-HU antibodies to detect retinal neurons and TUNEL to label apoptotic cells. Cells positive for both HU and TUNEL are indicated with arrows and cells positive only for TUNEL are labelled with arrowheads. Images were obtained from retinal sections of 10 embryos and representative overlay images are shown.

To determine if genetic loss of p53 can rescue excessive apoptosis in the *shh* ^−/−^ mutant, *p53* ^−/−^ *shh* ^−/−^ double mutant animals were generated using the previously published *tp53* ^M214K^ mutant zebrafish [44]. Indeed, high level of apoptosis in the *shh* ^−/−^mutants at 24 hpf (Fig. 3A-b) and in the retina at 72 hpf (Fig. 3B-b) compared to wild-type (Fig. 3A-a, 3B-a) was reduced in the *p53* ^−/−^ *shh* ^−/−^ mutant (Fig. 3A-c) to the wild-type level (Fig. 3A-c, 3B-c). I further examined differentiation status of apoptotic cells in the wild-type and *shh* ^−/−^ mutant retinas at 72 hpf by staining cells for neuronal marker HuC and apoptotic cells using TdT-mediated dUTP-biotin nick end labelling (TUNEL). In the wild-type retina, both HU-positive and negative apoptotic were present (Fig. 3C-a). By contrast, most apoptotic cells in the *shh* ^−/−^ mutant retina were undifferentiated (HuC-negative) (Fig. 3C-b), but some were HU-positive (Fig. 3C-b) suggesting that differentiating cells also die by apoptosis in the *shh* ^−/−^ mutant retina.

### PG13p21::nlsEGFP, a p53 reporter in the living zebrafish embryos

**Figure 4:**
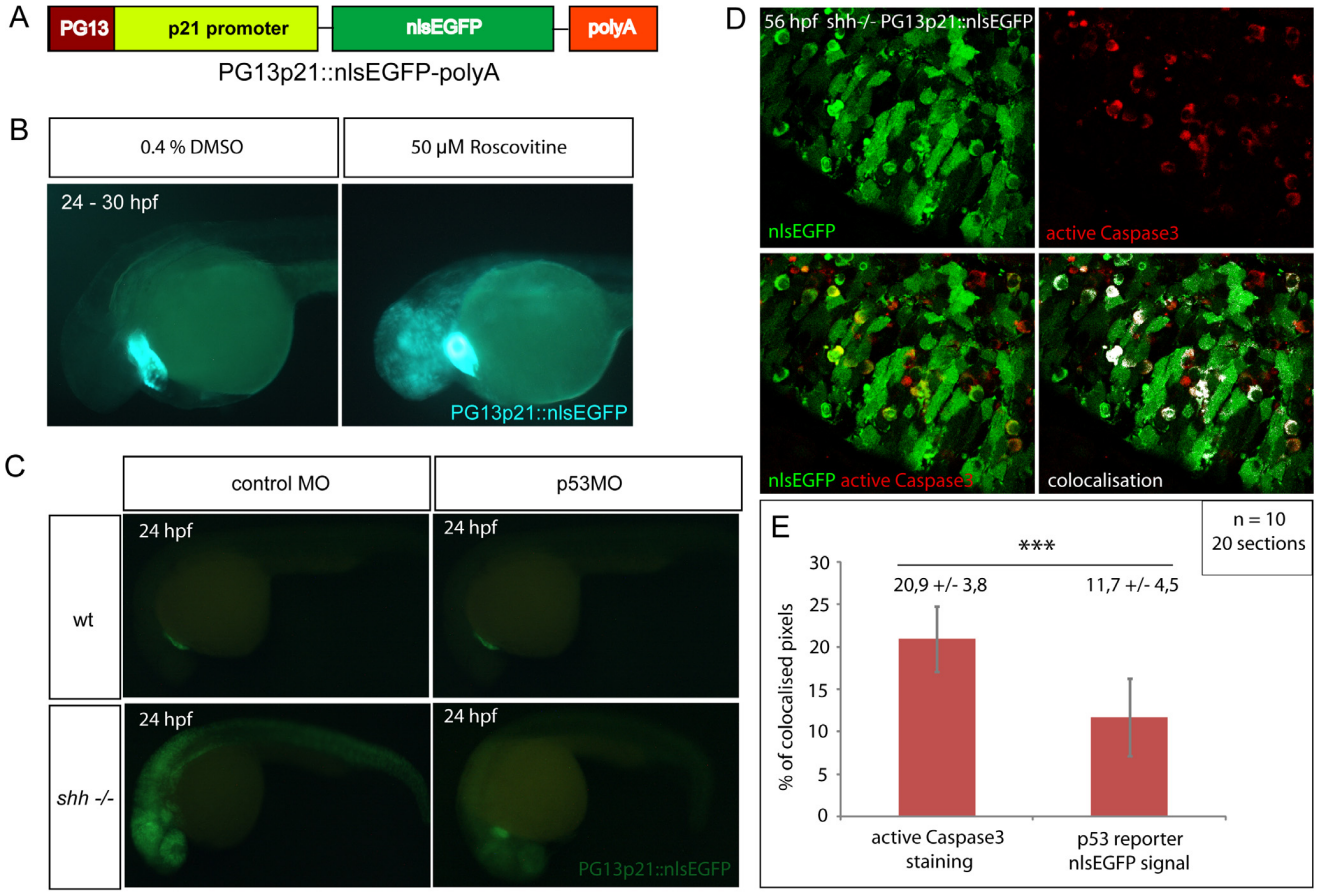
Zebrafish p53 reporter line construction and verification. (A) p53 reporter PG13p21::nlsEGFP-polyA was constructed using Tol2kit as described in Materials and methods. It consists of the p21 promoter fused with p53-binding enhancer sequence PG13, nlsEGFP coding sequence and a polyadenylation signal (polyA). (B) Wild-type embryos carrying the p53 reporter transgene were incubated for 6 hours at 24 hpf with either 50 µM Roscovitine or 0.4% DMSO diluted in E3 medium. DMSO-treated embryos did not show any significant expression of the reporter (right), whereas roscovitine-treated ones expressed p53 reporter at a high level (left). All embryos showed green-heart transgenesis marker. The experiment was repeated three times and 20 embryos were used for each treatment. (C) p53 morpholino but not 4-mismatch control morpholino injection suppressed expression of PG13p21::nlsEGFP p53 reporter in *shh* ^−/−^ mutant embryos at 24 hpf. Both injections had no effect on p53 reporter expression in the wild-type embryos. (D) Anti-active Caspase3 staining of cells in the *shh* ^−/−^ mutant retina at 56 hpf expressing p53 reporter transgene PG13p21::nlsEGFP. Most cells positive for active Caspase3 also expressed p53 reporter transgene. Single channel, overlay and co-localisation analysis images are shown. (E) Quantification of co-localisation of active Caspase3 staining and nlsEGFP p53 reporter signal. Co-localised pixels made up 20,9 +/− 3,8% of total active Caspase3 pixels and 11,7 +/− 4,5% of total nlsEGFP pixels. Quantifications were performed on 20 retinal section images from 10 embryos. Asterisks (***) indicate a significant statistical difference between the proportions (t-test, P-value<0,001).

To study the behaviour and characteristics of cells which have undergone activation of p53, I constructed a transgenic reporter to monitor p53 activity using a strategy similar to the one used to construct a p53 reporter in human cell lines [45]. A 2,4 kb promoter fragment of human *CDKN1A* encoding p21 CDK inhibitor and 13 copies of the optimal p53 binding site (PG13) [46] were combined into a p53-sensitive promoter. Tol2kit transgenic system [47] was then used to assemble the final construct containing PG13p21 promoter, nlsEGFP coding sequence and a polyA signal (Fig. 4A).

The p53 reporter zebrafish transgenic line is a general tool to study p53 activation and regulation. Studies of p53 in zebrafish employed camptothecin, a genotoxic drug, or roscovitine, a CDK inhibitor, to induce p53-dependent apoptosis [42], [48]. To verify that p53 reporter line can be used as a *bona fide* p53 reporter *in vivo*, I have treated p53 reporter transgenic fish with roscovitin. Wild-type embryos carrying the p53 reporter transgene were incubated either with 50 µM roscovitine or 0.4% DMSO as a control for 6 hours starting at 24 hpf. p53 reporter expression was strongly up-regulated in the brain and retina of roscovitine-treated but not in control DMSO-treated embryos (Fig. 4B). Furthermore, knocking down *p53* with morpholino in *shh* ^−/−^ mutant strongly inhibited expression of p53 reporter transgene at 24 hpf (Fig. 4C). Moreover, in the *shh* ^−/−^ mutant retina at 56 hpf, most apoptotic cells showed co-localisation of active Caspase3 labeling with p53 reporter nlsEGFP in the cytoplasm (Fig. 4D) suggesting that apoptosis is induced in cells with active p53. Co-localised pixels made 20,9 +/− 3,8% of total active Caspase 3 pixels, but only 11,7 +/− 4,5% of total p53 reporter nlsEGFP pixels, the difference being significant (t-test, P-value<0,001), (Fig. 4E) suggesting that apoptotic cells expressing p53 reporter are more frequent than p53 reporter-positive cells undergoing apoptosis, consistent with p53 activating caspases as a downstream result of its activity.

### Expression of p53 reporter in tissues requiring Shh for survival

**Figure 5:**
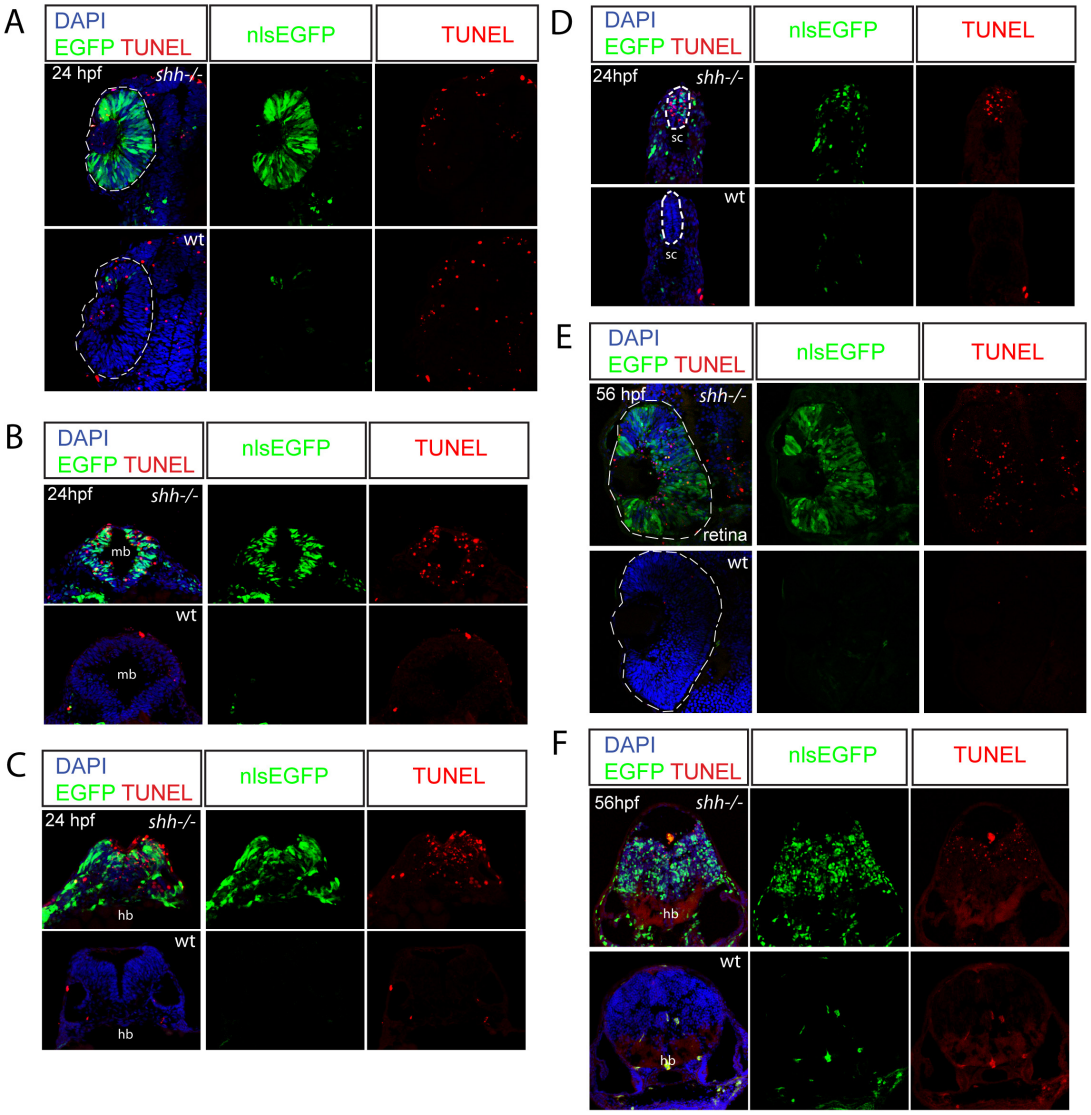
Expression of p53 reporter and apoptosis are strongly elevated in the *shh* ^−/−^ mutant embryos. Wild-type and *shh* ^−/−^ embryos at 24hpf (A–D) and 56 hpf (E, F) carrying p53 reporter transgene (PG13p21::nlsEGFP) were transversally sectioned, nuclei were stained with DAPI and apoptotic cells – with TUNEL, nlsEGFP signal is endogeneous (A–F). The developing nervous system of *shh* ^−/−^ mutant embryos showed high level of p53 reporter expression and apoptosis compared to wild-type embryos at both 24 (B–D) and 56 hpf (F). (A) At 24 hpf, p53 reporter was strongly induced in the *shh* ^−/−^ mutant compared to the wild-type retina, but there was no elevated apoptosis in the *shh* ^−/−^ mutant retina. (B) Expression of p53 reporter and TUNEL labeling in midbrain of *shh* ^−/−^ mutant and wild-type embryos at 24 hpf. (C) Expression of p53 reporter and TUNEL labeling in hindbrain of *shh* ^−/−^ mutant and wild-type embryos at 24 hpf. (D) Expression of p53 reporter and TUNEL labelling in spinal cord of *shh* ^−/−^ and wild-type at 24 hpf. (E) At 56 hpf, both p53 reporter expression and apoptosis were much higher in the *shh* ^−/−^ retina than in the wild-type retina. (F) Expression of p53 reporter and TUNEL labelling in hindbrain regions of *shh* ^−/−^ and wild-type embryos at 56 hpf.

I have analysed p53 reporter expression at 24 hpf and 56 hpf in the *shh* ^−/−^ mutant and wild-type embryos (Fig. 5). At 24 hpf, *shh* ^−/−^ mutants expressed much higher level of nlsEGFP and contained many more TUNEL-positive cells in midbrain, hindbrain and spinal cord than wild-type embryos (Fig. 5B, C, D). I also detected that at 56 hpf *shh* ^−/−^ mutant hindbrain expressed the p53 reporter at a higher level than wild-type embryos (Fig. 5F). By contrast, *shh* ^−/−^ mutant retina showed strong expression of the p53 reporter but no elevated apoptosis at 24 hpf (Fig. 5A). This result is interesting and unexpected as *shh* expression starts in the retina at 28 hpf [49]. However, at 56 hpf in the *shh* ^−/−^ mutant retina, both p53 reporter expression and number of TUNEL-positive cells are high compared to wild-type (Fig. 5E). Activation of p53 in *shh* ^−/−^ mutant retinal cells before 28 hpf indicates that they require Shh signalling at an earlier stage to control p53 activity. In the absence of Shh, neural crest cells undergo apoptosis [10] and indeed some p53 reporter positive cells present in the midbrain are located in a region populated by neural crest cells (Fig. 5B). p53 reporter also labeled somite cells (Fig. 5D), which are likely sclerotome cells requiring Shh for their survival [7].

### Hh signalling activation by dnPKA-GFP decreases *cyclinG1* expression and apoptosis in *shh* ^−/−^ mutant embryos at 12-somite stage

**Figure 6:**
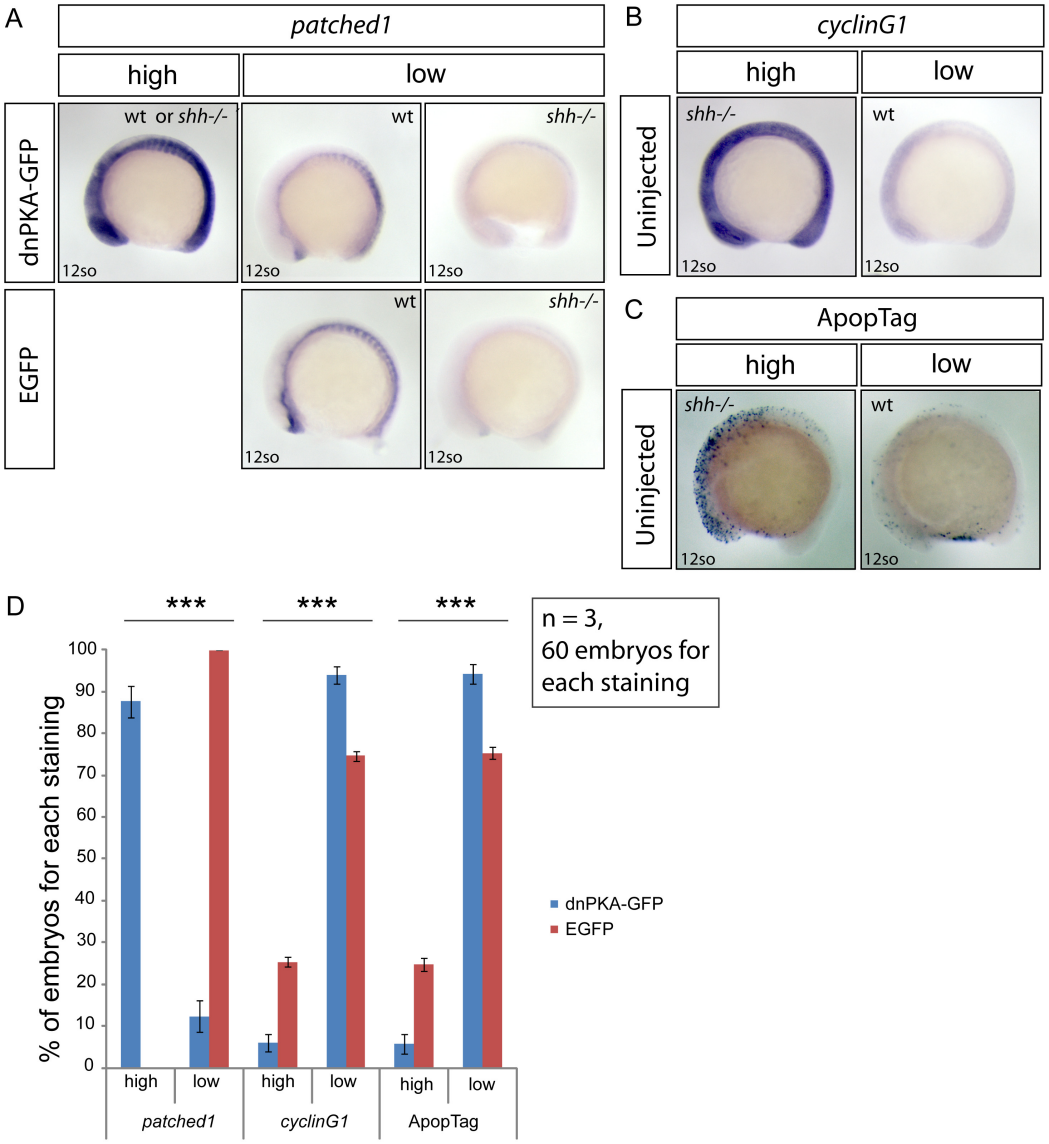
Hh signaling activation by dnPKA-GFP decreases p53 target *cyclinG1* expression and suppresses apoptosis in *shh* ^−/−^ mutant embryos. Embryos from *shh* ^+/–^ parent fish were injected with either dnPKA-GFP or EGFP mRNA, grown to 12-somite stage and *in situ* stained for *patched1*, target gene of Hh signaling, and *cyclinG1*, p53 target gene, and ApopTag staining was performed to characterize the level of apoptosis. (A) Injection of dnPKA-GFP led to a strongly increased and more widely spread expression of *patched1* (category “high”) in 87,7+/−3,8% of injected embryos, the rest having normal wild-type or low *shh* ^−/−^ mutant *patched1* expression comparable to expression of *patched1* in EGFP-injected embryos from *shh* ^+/–^ parent fish (category “low”). (B) Expression of *cyclinG1* in uninjected embryos from *shh* ^+/–^ parent fish. Categories “high” and “low” indicate expression levels in *shh* ^−/−^ mutant and wild-type embryos, respectively. (C) ApopTag staining of apoptotic cells in uninjected embryos from *shh* ^+/–^ parent fish. Categories “high” and “low” indicate levels of apoptosis in *shh* ^−/−^ mutant and wild-type embryos, respectively. (D) Statistical analysis of results from injections of dnPKA-GFP and EGFP mRNA into embryos from *shh* ^+/–^ parent fish. All of EGFP-injected embryos had “low” expression of *patched1*, 25,3 +/− 1,15% (Mean +/− standard deviation) of them had “high” expression of *cyclinG1* and 24,7 +/− 1,53% had “high” apoptosis levels. dnPKA-GFP mRNA injection led to “high” expression of *patched1* in 87,7 +/− 3,8% of embryos and decreased expression of *cyclinG1* and apoptosis levels: “high” proportions are 6 +/− 2% and 5,8 +/− 2,4%, respectively. The experiment was done 3 times and 60 embryos were used for each staining. Asterisks (***) on top of *shh* ^−/−^ mutant bars indicate significant statistical differences of proportions (Fisher's exact test, P-value<0,001).

In tissue culture experiments, p53 activation has been shown to be inhibited by Gli-mediated activation of an unknown p53 pathway inhibitor [18]. This leads to the question whether activation of Hh signalling inhibits p53 pathway *in vivo* in the absence of Shh. To activate the canonical Hh/Gli signaling pathway, I injected mRNA of dominant-negative PKA fused with GFP (dnPKA-GFP) [50], [51] or EGFP mRNA as a control into progeny of fish heterozygous for the *shh* deletion. Given the limited mRNA stability, I analysed the effects of RNA injections at the 12 somites stage, when *shh* ^−/−^ mutant embryos already show a higher level of p53 target gene expression and apoptosis than wild-type embryos (Fig. S2). While all the EGFP-injected embryos showed unaltered wild-type or *shh* ^−/−^ mutant *patched1* expression pattern (Fig. 6A, D, “low”), over-expression of dnPKA-GFP led to ectopic spreading of *patched1* expression in 87,7 +/− 3,8% of embryos (Fig. 6A, D, “high”). I next assessed expression of *cyclinG1*, p53 target gene, after mRNA injections by visually scoring expression strength as “high” or “low” based on staining of uninjected embryos (Fig. 6B), where embryos with “high” *cyclinG1* expression were inferred to be *shh* ^−/−^ mutant and those with “low” expression were wild-type (control stainings of wild-type embryos, not shown). In this experiment, the effect of dnPKA-GFP on p53 target gene expression is inferred from the change in proportion of embryos with stronger *cyclinG1* expression. While after EGFP mRNA injection the proportion of embryos with “high” expression of *cyclinG1* was 25,3 +/− 1,2% (Fig. 6D), only 6 +/− 2% (Fig. 6D) of dnPKA-GFP -injected embryos had perceptibly stronger *cyclinG1* expression than the other embryos in the samples (Fig. 6D). Overexpression of dnPKA-GFP also reduced the proportion of embryos with higher apoptosis levels (Fig. 6C) to 5,8 +/− 2,4% (Fig. 6D) (Fig. 6D) compared to 24,7 +/− 1,53% (Fig. 6D) among EGFP-injected embryos (Fig. 6D). Taken together, these data indicate that activation of Hh signalling using dnPKA-GFP can suppress p53 target expression and apoptosis.

### p53 regulates the mitotic indices in the *shh* ^−/−^ mutant retina

**Figure 7:**
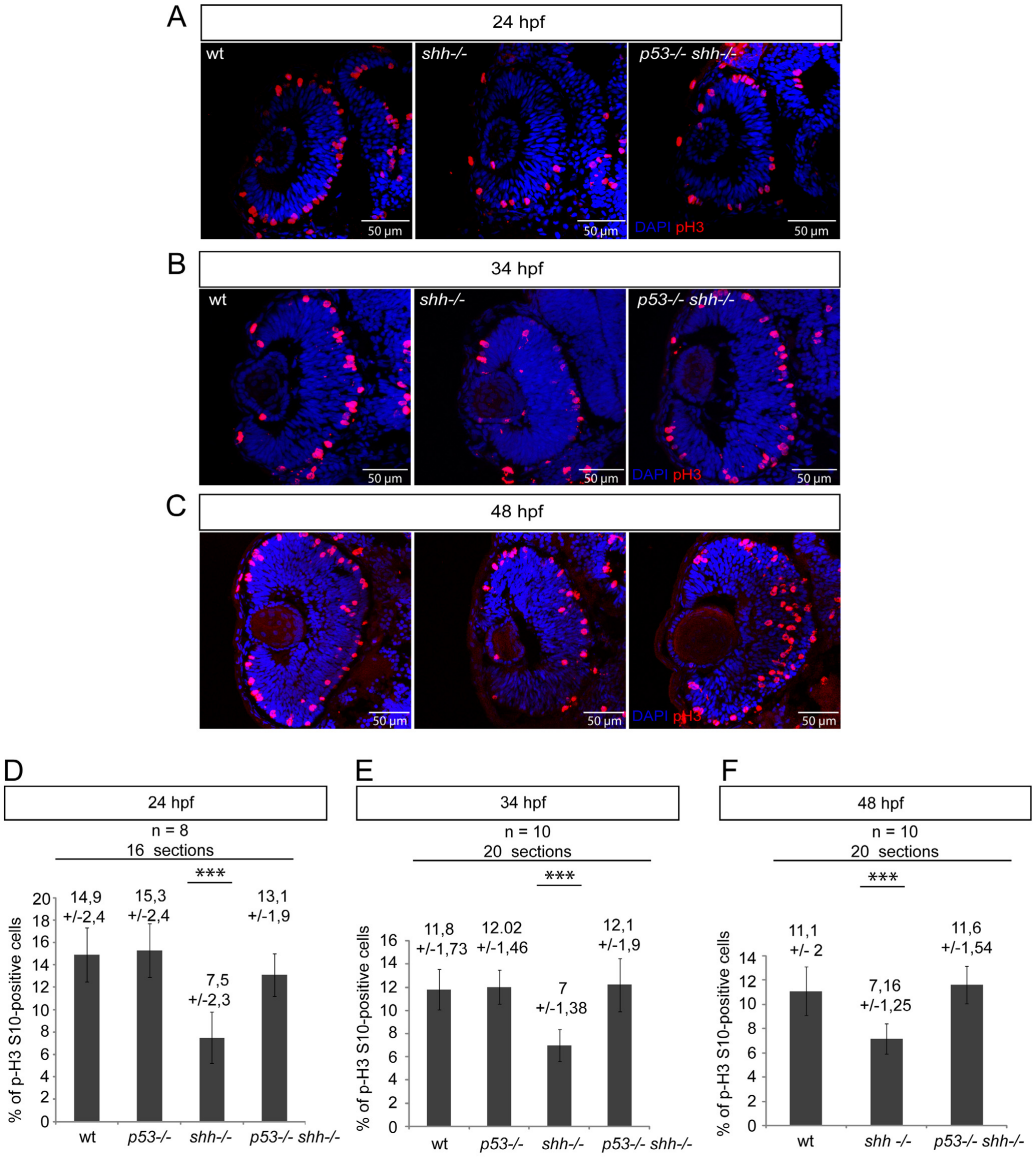
p53 regulates the mitotic index in the *shh* ^−/−^ mutant retina during early progenitor proliferation and neurogenesis. pH 3/DAPI staining of wild-type, *shh* ^−/−^ and *p53* ^−/−^ *shh* ^−/−^ retinas was done at 24 hpf (A), 34 hpf (B) and 48 hpf (C). At all stages, the mitotic index in the *shh* ^−/−^ retina was lower than in the wild-type retina, while in the *p53* ^−/−^ *shh* ^−/−^ retina, the mitotic index was comparable to that of the wild-type retina. (D) Statistical analysis of mitotic indices in wild-type, *p53* ^−/−^, *shh* ^−/−^ and *p53* ^−/−^ *shh* ^−/−^ retinas at 24 hpf, the indices being comparable in wild-type (14,9%, SD = 2,4%), *p53* ^−/−^ (15,3%, SD = 2,4%), and *p53* ^−/−^ *shh* ^−/−^ (13,1%, SD = 1,9%) embryos and significantly higher than in the *shh* ^−/−^ mutant retina (7,5%, SD = 2,3%). (E) Statistical analysis of mitotic indices in the wild-type, *p53* ^−/−^, *shh* ^−/−^ and *p53* ^−/−^ *shh* ^−/−^ retinas at 34 hpf, comparable in wild-type (11,8%, SD = 1,73%), *p53* ^−/−^ (12,02%, SD = 1,46%), and *p53* ^−/−^ *shh* ^−/−^ (12,1%, SD = 1,9%) retinas, whereas the mitotic index in the *shh* ^−/−^ mutant retina was almost twice lower (7%, SD = 1,38%). (F) Statistical analysis of mitotic indices in wild-type, *shh* ^−/−^ and *p53* ^−/−^ *shh* ^−/−^ retinas at 48 hpf, the indices being comparable in wild-type (11,1%, SD = 2%) and *p53* ^−/−^ *shh* ^−/−^ (11,6%, SD = 1,54%) embryos and significantly higher than in the *shh* ^−/−^ mutant retina (7,16%, SD = 1,25%). Either 8 (D) or 10 (E, F) embryos (2 retinal sections per embryo) were analysed for each genotype and mean +/− standard deviation indicated above the bars of mitotic index statistics. Asterisks (***) on top of *shh* ^−/−^ mutant bars indicate their significant statistical differences from other samples (t-test, P-value<0,001).

To determine the effect of p53 loss on proliferation in the absence of Shh, I quantified mitotic cells in the wild-type, *p53* ^−/−^, *shh* ^−/−^ and *p53* ^−/−^ *shh* ^−/−^ mutant retinas at 24, 34 and 48 hpf stages by staining retinal sections for Ser10-phosphorylated form of Histone3 (pH 3), a reliable marker for the late G2-phase and mitosis [52]. At 24 hpf, retinal cells in zebrafish are highly proliferative and are not exiting the cell cycle, while at 34 and 48 hpf, retinal cells are exiting the cell cycle and acquire terminal fates. Analysis of how p53 affects proliferation in the *shh* ^−/−^ mutant retina at 24 hpf and during neurogenesis will shed light on the role of p53 in early progenitor proliferation and their cell cycle exit. Retinal mitotic indices at 24 hpf in wild-type (14,9 +/− 2,4%) (Mean +/− standard deviation), *p53* ^−/−^ (15,3 +/− 2,4%) and *p53* ^−/−^ *shh* ^−/−^ (13,1 +/− 1,9%) were pairwise not significantly different (P-value>0.1), but differed significantly from the mitotic index of *shh* ^−/−^ mutant (7,5 +/− 2,3%) (Fig. 7A, D), suggesting that *p53* loss rescues early retinal progenitor proliferation to a nearly normal level. At both 34 and 48 hpf, I found much fewer mitotic cells in the *shh* ^−/−^ mutant than in the wild-type retina (Fig. 7B, C). Strikingly, the *p53* ^−/−^ *shh* ^−/−^ mutant retina contained more mitotic cells and was larger than the *shh* ^−/−^ mutant retina at both 34 and 48 hpf (Fig. 7B, C). At 34 hpf, mitotic indices in the wild-type retina (11,8 +/− 1,73%) and in the *p53* ^−/−^ mutant retina (12 +/− 1,46%) were not statistically different (t-test, P = 0,67) suggesting that *p53* loss alone does not increase the mitotic index (Fig. 7E). By contrast, the mitotic index in the *shh* ^−/−^ mutant retina (7 +/− 1,38%) was significantly lower than in the wild-type retina or in the *p53* ^−/−^ *shh* ^−/−^ retina (12,1 +/− 1,9%) (t-test, P<0,001) (Fig. 7E), not different from the wild-type mitotic index (t-test, P = 0,55) (Fig. 7E). At 48 hpf, the mitotic indices in the wild-type (11,1 +/− 2%) and *shh* ^−/−^ mutant (7,16 +/− 1,25%) retinas were significantly different (t-test, P<0,001) (Fig. 7F), and there was no difference between mitotic indices of the *p53* ^−/−^ *shh* ^−/−^ mutant retina (11,6 +/− 1,54%) and the wild-type retina (t-test, P =  0,34) (Fig. 7F). Thus, the results at 24, 34 and 48 hpf conclusively demonstrate that p53 decreases mitotic indices in the *shh* ^−/−^ mutant retina during early retinal progenitor proliferation and neurogenesis.

### *p53* inhibits cell-cycle exit in the *shh* ^−/−^ mutant retina

**Figure 8:**
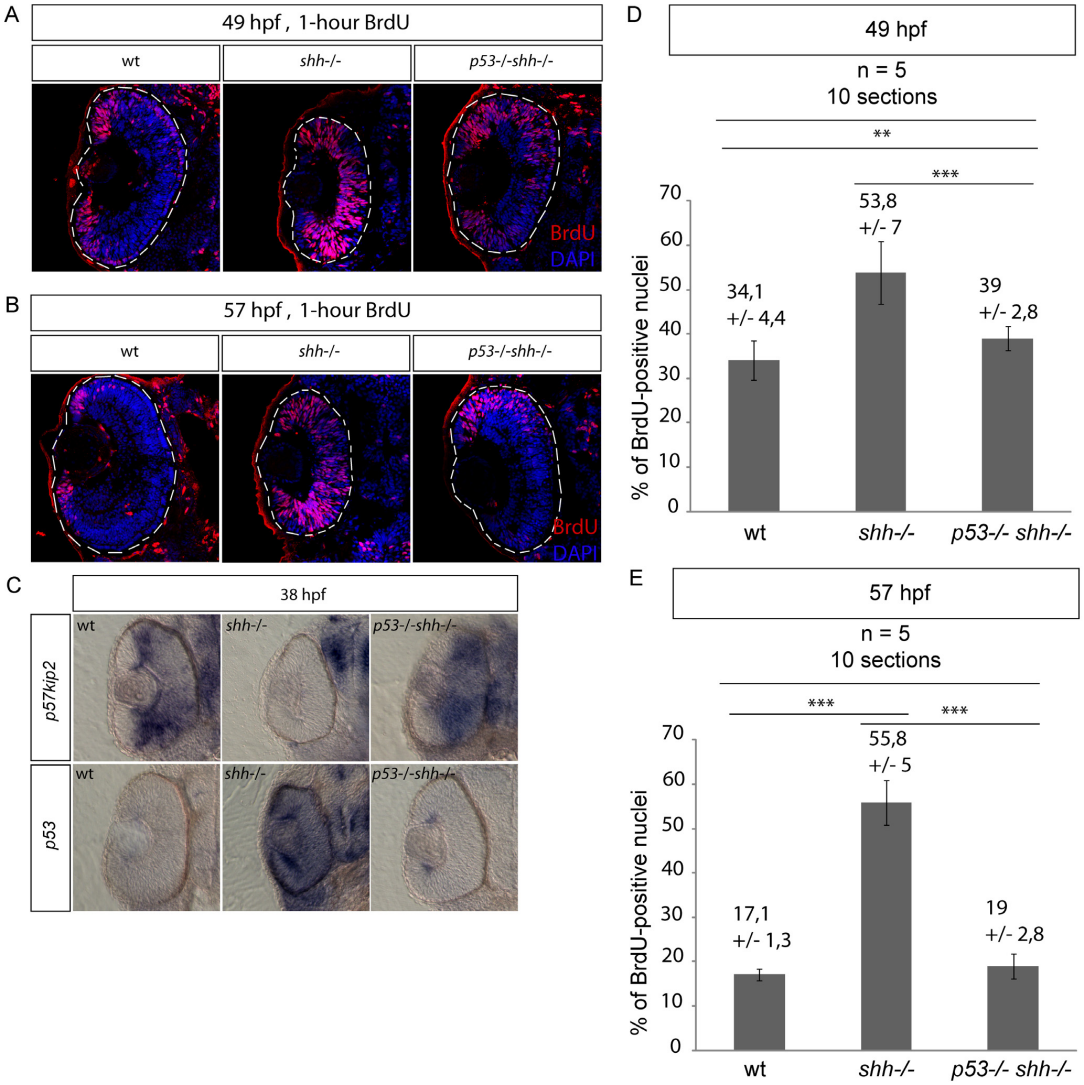
Loss of *p53* rescues cell-cycle exit defect in the *shh* ^−/−^ mutant retina. S-phase progression in wild-type, *shh* ^−/−^ and *p53* ^−/−^ *shh* ^−/−^ mutants was analysed at 49 and 57 hpg after one-hour labelling with BrdU. Confocal sections stained with anti-BrdU antibodies and DAPI are shown with the anterior side to the top. (A) Anti-BrdU/DAPI staining of wild-type, *shh* ^−/−^ and *p53* ^−/−^ *shh* ^−/−^ mutant retinas at 49 hpf identified a greater extent of cell-cycle exit (fewer cells labelled) in the wild-type and *p53* ^−/−^ *shh* ^−/−^ mutant than in the *shh* ^−/−^ retina. The data from these stainings were then used for quantification in (D). (B) At 57 hpf in the wild-type retina, BrdU-positive cells were found only in the ciliary marginal zone (CMZ). However, in the *shh* ^−/−^ mutant retina, cells still failed to exit the cell cycle. Like in the wild-type retina, cells of *p53* ^−/−^ *shh* ^−/−^ mutant mostly exited the cell cycle and proliferative cells were located only in the CMZ. (C) At 38 hpf, *p57kip2* expression was at a high level in both wild-type and *p53* ^−/−^ *shh* ^−/−^ mutant retinas, whereas no *p57kip2* was present in the *shh* ^−/−^ mutant retina. By contrast, *p53* expression was high in the *shh* ^−/−^ mutant retina and very low in the wild-type and *p53* ^−/−^ *shh* ^−/−^ mutant retinas. (D) Proportions of BrdU-positive cells (S-phase indices) in wild-type, *shh* ^−/−^ and *p53* ^−/−^ *shh* ^−/−^ retinas at 49 hpf were obtained from confocal sections such as presented in (A). The S-phase indices in wild-type (34,1%, SD = 4,4%) and *p53* ^−/−^ *shh* ^−/−^ (39%, SD = 2,8%) were each significantly lower than in the *shh* ^−/−^ mutant retina (53,8%, SD = 7%) (t-test, P-value<0,001), the difference between them still being significant (t-test, P-value<0,01). (E) S-phase indices in wild-type, *shh* ^−/−^ and *p53* ^−/−^ *shh* ^−/−^ retinas at 57 hpf were obtained from confocal sections such as presented in (B). The S-phase indices in wild-type (17,1%, SD = 1,3%) and *p53* ^−/−^ *shh* ^−/−^ (19%, SD = 2,8%) were not significantly different (t-test, P-value>0,05), but were each significantly lower than in the *shh* ^−/−^ mutant retina (55,8%, SD = 5%) (t-test, P-value<0,001). Total 10 sections from 5 embryos (2 retinal sections per embryo) were analysed for each genotype and mean +/− standard deviation are indicated above the graph bars. Statistical differences were evaluated using t-test with P-values<0,01 (**) or<0,001 (***).

The normal mitotic index during neurogenesis in the *p53* ^−/−^ *shh* ^−/−^ retina could result from the rescue of cell-cycle exit of retinal progenitors by p53 loss. On the other hand, failure of cell-cycle exit could activate p53, even though p53 activation occurs much earlier than cell-cycle exit in the *shh* ^−/−^ mutant retina (Figs. 5, S2). To distinguish between these scenarios, I directly analyzed cell-cycle exit progression using one-hour 5-bromo-deoxyuridine (BrdU) incorporation in the wild-type, *shh* ^−/−^ and *p53* ^−/−^ *shh* ^−/−^ mutant retinas at 48 and 56 hpf stages, when cell-cycle exit in the retina is being completed, with subsequent quantification of BrdU-positive cells using 4′,6-Diamidino-2-phenylindol (DAPI) staining. At 49 hpf, the proportion of BrdU-positive cells in the *shh* ^−/−^ retina (53,8 +/− 7%) was significantly higher (t-test, P-value<0,001) than in either the wild-type (34,1 +/− 4,4%) or *p53* ^−/−^ *shh* ^−/−^ retinas (39 +/− 2,8%), the difference between wild-type and *p53* ^−/−^ *shh* ^−/−^ BrdU-positive proportions still being significant (t-test, P-value<0,01) (Figs. 8A, D), suggesting a slight delay of cell-cycle exit in the *p53* ^−/−^ *shh* ^−/−^ retina compared to wild-type. By 57 hpf, cell-cycle exit in the retina progressed decreasing the proportions of BrdU-positive cells in wild-type (17,1 +/− 1,3%) and *p53* ^−/−^ *shh* ^−/−^ (19 +/− 2,8%) embryos, which were both significantly lower than in the *shh* ^−/−^ retina (55,8 +/− 5%) (t-test, P-value<0,001). Interestingly, at 57 hpf BrdU the proportions of BrdU-positive cells in wild-type and *p53* ^−/−^ *shh* ^−/−^ retinas were not different (t-test, P-value>0,05), suggesting a complete rescue of cell-cycle exit in the *shh* ^−/−^ retina by *p53* loss.

I then addressed the hypothesis that *p53* loss in the *shh* ^−/−^ retina induces expression of *p57kip2*, a cyclin-dependent kinase inhibitor essential for cell-cycle exit in the zebrafish retina. Indeed, at 38 hpf, *p57kip2* was expressed in large areas of wild-type and *p53* ^−/−^ *shh* ^−/−^ mutant retinas, but absent from the *shh* ^−/−^ mutant retina (Fig. 8C), whereas *p53* was much more strongly expressed in the *shh* ^−/−^ mutant retina than in the wild-type and *p53* ^−/−^ *shh* ^−/−^ mutant retinas (Fig. 8C). Altogether, these results suggest that the delay of cell-cycle exit in the *shh* ^−/−^ mutant retina is dependent on the p53 activity inhibiting expression of the CDK inhibitor p57kip2.

### *p53* loss rescues amacrine and photoreceptor cells in the *shh* ^−/−^ mutant retina

**Figure 9:**
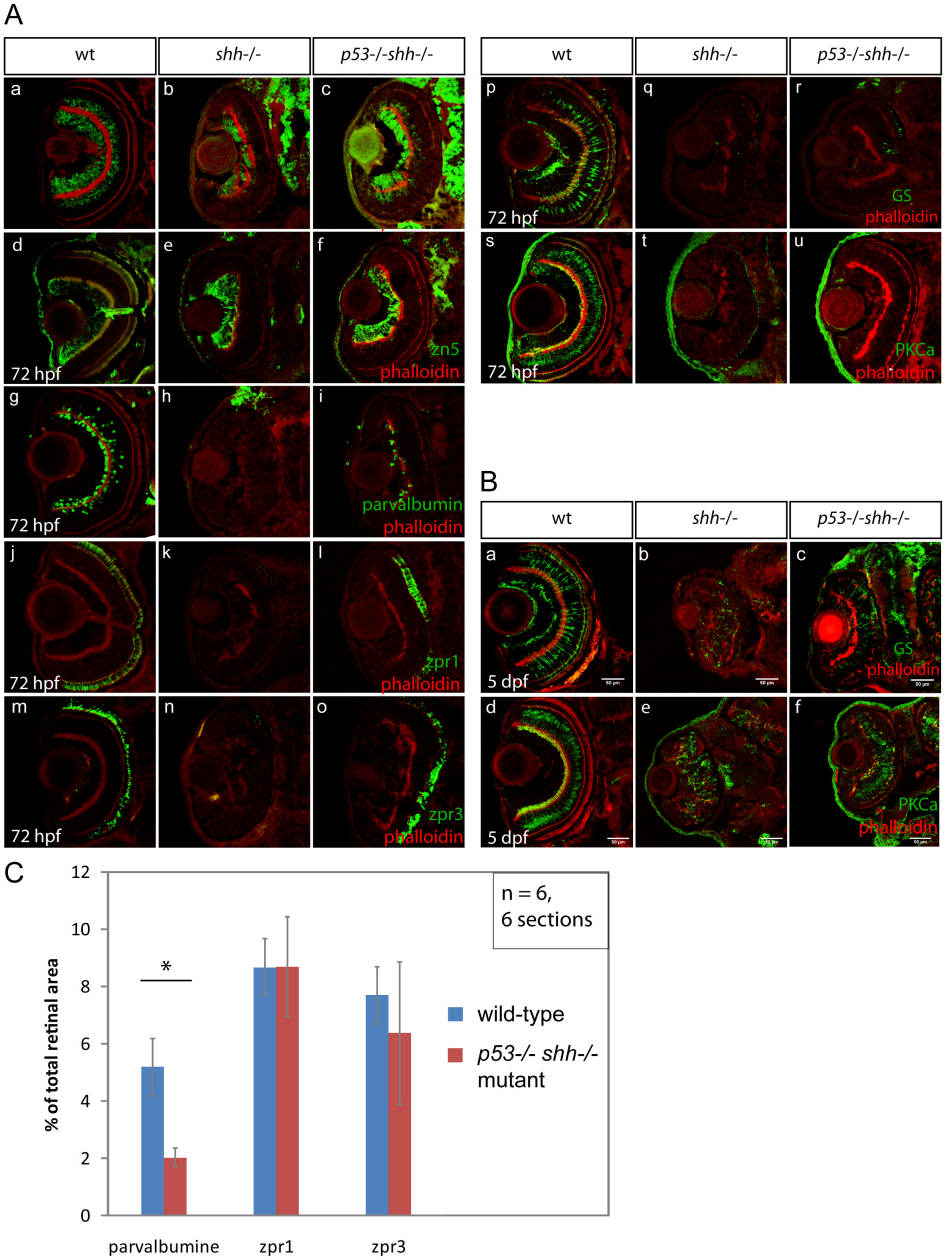
*p53* loss rescues amacrine and photoreceptor cells in the *shh* ^−/−^ mutant retina. Differentiation of retinal cell types was assessed using specific antibodies (A, B). The sections are oriented with their anterior side to the top. All sections were stained with phalloidin-Alexa568 (A, a–u; B, a–f) to visualize retinal lamination. Staining for HuC neuronal antigen labelled ganglion and amacrine cells in the wild-type retina (A, a), ganglion and very few amacrine cells in the *shh* ^−/−^ mutant retina (A, b). In the *p53* ^−/−^ *shh* ^−/−^ retina, however, labelling of both ganglion cells and amacrine cells was partially restored (A, c). Ganglion cells labelled by an anti-zn5 antibody were present in comparable numbers in wild-type (A, d), *shh* ^−/−^ mutant (A, e) and *p53* ^−/−^ *shh* ^−/−^ mutant retinas (A, f). Amacrine cells labelled by anti-parvalbumine antibody were present in the wild-type retina (A, g), absent in the *shh* ^−/−^ mutant (A, h) and partially rescued in the *p53* ^−/−^ *shh* ^−/−^ mutant retina (A, i). Red-green double cones (zpr1 antibody labelling) and rod photoreceptors (zpr3 antibody labeling) were present in the wild-type retina (A, j, m), absent in the *shh* ^−/−^ mutant retina (A, k, n) and rescued in the *p53* ^−/−^ *shh* ^−/−^ mutant retina (A, l, o). Staining for glutamine synthetase (GS) labelled Müller glia cells in wild-type embryos (A, p). In both *shh* ^−/−^ and *p53* ^−/−^ *shh* ^−/−^ retinas, very few Müller glia were present (A, q, r). Bipolar cells were labelled in the wild-type retina by anti-PKCα antibody staining (A, s), but absent in *shh* ^−/−^ and *p53* ^−/−^ *shh* ^−/−^ retinas (A, t, u). At 5 dpf, Müller glia and bipolar cells were detected in *shh* ^−/−^ and *p53* ^−/−^ *shh* ^−/−^ retinas (B, b,c,e,f) but in lower numbers than in the wild-type retina (B, a,d). All stainings were performed on at least 6 embryos for each genotype and representative images are shown. (C) Quantitation of rescue of amacrine cells (parvalbumine labelling), red-green double cone (zpr1) and rod photoreceptors (zpr3) in the *p53* ^−/−^ *shh* ^−/−^ mutant retina by plotting ratios of segmented area for each staining to the total retina area. Amacrine cells occupy a significantly larger relative area in the wild-type than in the *p53* ^−/−^ *shh* ^−/−^ mutant retina (* on top of the bars, t-test, P-value<0,05), whereas relative areas of both types of photoreceptors are not significantly different in wild-type and *p53* ^−/−^ *shh* ^−/−^ mutant retinas.

To address the question whether p53 affects differentiation in the *shh* ^−/−^ mutant, retinal sections of wild-type, *shh* ^−/−^ and *p53* ^−/−^ *shh* ^−/−^ mutant embryos were stained for markers of retinal cell types. Staining for HuC neuronal marker strongly labelled both ganglion and amacrine cells in the wild-type (Fig. 9A-a), but only ganglion cells in the *shh* ^−/−^ mutant retina (Fig. 9A-b), whereas in the *p53* ^−/−^ *shh* ^−/−^ mutant retina, both ganglion and amacrine cells were labelled (Fig. 9A-c). Consistently, retinal ganglion cells (RGCs) identified by anti-zn5 staining were present in retinas of all three genotypes (Fig. 9A-d, e, f). However, amacrine cells labelled by anti-parvalbumin antibodies in the wild-type retina (Fig. 9A-g), were absent in the *shh* ^−/−^ retina (Fig. 9A-h) and partially rescued in *p53* ^−/−^ *shh* ^−/−^ embryos (Fig. 9A-i, C). The ratio of area covered by parvalbumine signal to the total retinal section area in the wild-type retina (5,19 +/− 1,6%) was significantly different from this ratio in the *p53* ^−/−^ *shh* ^−/−^ retina (2,03 +/− 0,32%), the efficiency of rescue being 39% (Fig. 9C). Investigating whether p53 affects differentiation of photoreceptors, it was found that red-green cone and rod photoreceptors present in the wild-type retina (Fig. 9A-j, m) are absent in the *shh* ^−/−^ retina (Fig. 9A-k, n) and rescued in the *p53* ^−/−^ *shh* ^−/−^ mutant retina (Fig. 9A-l, o), the rescue being complete since the relative areas of photoreceptor labeling were not different between retinas of wild-type and *p53* ^−/−^ *shh* ^−/−^ embryos (Fig. 9C). Müller glia, a cell type required to maintain the retinal environment, was present in the wild-type retina at 72 hpf (Fig. 9A-p) but nearly absent in both *shh* ^−/−^ (Fig. 9A-q) and *p53* ^−/−^ *shh* ^−/−^ mutant retinas (Fig. 9A-r). Bipolar cells, a retinal cell type involved in signal transduction during light perception, were labelled by staining for PKCα in the wild-type retina at 72 hpf (Fig. 9A-s), but were absent in both *shh* ^−/−^ and *p53* ^−/−^ *shh* ^−/−^ mutant retinas (Fig. 9A-t, u). However, at 5 days post fertilization (dpf), Müller glia and bipolar cells appeared in both *shh* ^−/−^ (Fig. 9B-b, e) and *p53* ^−/−^ *shh* ^−/−^ (Fig. 9B-c, f) mutant retinas at similar levels indicating that their differentiation is strongly delayed in the absence of Shh and is not affected by p53 loss. As control, I checked whether loss of *p53* alone affects retinal differentiation and found that differentiation of retinal cell types in wild-type and *p53* ^−/−^ mutant embryos was comparable (Fig. S3). These results suggest that p53 inhibits differentiation of amacrine cells and photoreceptors in the *shh* ^−/−^ mutant, without affecting differentiation of other cell types.

## Discussion

### p53 is essential for elevated apoptosis in the absence of Shh

In vertebrates, Shh signalling is crucial to prevent cell death in many different contexts, but the mechanism of Shh-mediated survival regulation is still unclear. To clarify this mechanism, I focused on apoptosis in the neural tube and retina of the *shh* ^−/−^ mutant.

I first examined Bcl2 family gene expression as a potential cause of apoptosis in the *shh* ^−/−^ mutant but haven't detected any differences between wild-type and *shh* ^−/−^ mutant embryos (Fig. S1). However, activation of Hh signalling has been shown to induce expression of *bcl2* gene in mouse keratinocytes [14], chick spinal cord [13] and to maintain *Bcl2* gene expression in the mouse lymphoma cells [53]. Thus, Hh signalling activates *Bcl2* expression in certain contexts, but my results suggest that Shh signaling is not required for global regulation of Bcl2 family genes during zebrafish development.

I then show that p53 activates expression of its target genes and induces apoptosis in the neural tube and retina in the absence of Shh (Figs. 1, 2). Over-expression of EGFP-bcl2 [54] suppressed elevated apoptosis in the *shh* ^−/−^ mutant showing that intrinsic apoptotic pathway is activated. I then determined that the onset of p53 pathway activation and elevated apoptosis in the *shh* ^−/−^ mutant happened at around 10somite stage (Fig. S2). The broad pattern of p53 target gene expression and apoptosis in the *shh* ^−/−^ mutant at 10somite stage argues against cell death due to differentiation failure, since few cells in the wild-type embryos express neuronal differentiation marker HuC at this stage [55]. Moreover, early requirement of Shh for cell survival during development is consistent with the observations that in the neural tube Hh signaling regulates cell survival independently from and earlier than its effects on cell fate [13].

### PG13p21::nlsEGFP transgenic line faithfully reports regions with p53 activation

To detect cells that have undergone p53 activation, I constructed the PG13p21::nlsEGFP transgenic p53 reporter. Expression of this reporter in *shh* ^−/−^ mutant was dependent on p53, inducible by roscovitine and co-localised with activeCaspase3 labeling in the *shh* ^−/−^ mutant at 56 hpf (Fig. 4) suggesting that the PG13p21::nlsEGFP line is a *bona fide* p53 reporter.

Expression of p53 reporter in the *shh* ^−/−^ mutant retina at 24 hpf was much higher than in the wild-type retina but few apoptotic cells were detected in both cases (Fig. 5), suggesting that extraretinal Shh controls p53 activity during early retinal development before the onset of Shh production in the retina at 28 hpf [49]. However, at 56 hpf, apoptotic cells and p53 reporter-positive cells are both present in the retina indicating a delay between initial p53 activation and apoptosis induction in the *shh* ^−/−^ mutant retina probably due to a high level of pro-survival factors in the early retina. Extraretinal Hh signalling is known to be required for early retinal patterning, for example, Stenkamp and Frey (2003) showed that inhibition of Hh signalling from 10 hpf reduces *ath5* retinal patterning gene expression stronger than the same treatments during retinal neurogenesis [56], and Kay and co-authors (2005) demonstrated that Hh signalling inhibition from 13 hpf but not from 25 hpf can delay expression of *ath5* [57]. Moreover, Masai et al. (2005) found using forskolin, an activator of PKA, that Hh signalling during 22–26 hpf period is essential for ath5 expression wave, also highlighting the role of extraretinal Hh signalling for retinal patterning [36]. My findings are consistent with these studies, and also implicate extraretinal Hh signalling in regulation of p53 activity in the retina.

In addition, I observed p53 reporter activation and apoptosis in the midbrain, hindbrain and spinal cord regions of the neural tube (Fig. 5), consistent with the previous studies on the survival function of Shh in these tissues [6], [9]. Although p53 reporter expression but no elevated apoptosis were detected in somites of the *shh* ^−/−^ mutant (Fig. 5D), p53 may mediate apoptosis in the somites of the *shh* ^−/−^ mutant at earlier stages, which would make the elevated p53 reporter expression fully consistent with the role of Shh in sclerotome cell survival and myogenic differentiation [7]. Locations of p53 reporter-positive cells in the lateral midbrain are suggestive of their neural crest origin (Fig. 5), agreeing with the requirement of Shh for their survival [10]. Taken together, these results show high expression of the p53 reporter transgenic reporter in the regions requiring Shh for cell survival and the advantages of p53 reporter over *in situ* hybridizations for p53 target genes or antibody stainings against p53 [48] thanks to the possibility to do live imaging, label specific cell parts and change the fluorescent protein used for labeling.

### Activation of Hh signalling suppresses p53 pathway in the *shh* ^−/−^ mutant

Activation of p53 in the absence of Shh raises the question whether this elevated p53 activity can be suppressed by Hh signaling activation. Indeed, Hh signaling activation using dnPKA-GFP effectively suppressed p53 target gene expression and apoptosis in the *shh* ^−/−^ mutant at 12somite stage (Fig. 6). While this study was in progress, Abe et al. (2008) have suggested that Hh signaling regulates stability of p53 by activating Mdm2 in human cell lines by inducing an unknown Mdm2-activating factor [18]. Stecca and Altaba (2009) characterized the interaction between p53 and Gli1, a downstream Hh signalling effector, in wild-type mouse neural stem cells [19]. These authors found that Gli1 can suppress p53 activity by up-regulating Mdm2 level. Since p53 activity can be inhibited by Hh signaling activation in human cell lines, mouse neural stem cells and zebrafish embryos, the same mechanism is likely involved.

Concerning p53 regulation, the question is whether Hh signaling is required for establishment or maintenance of p53 activity control. Given the localized nature of Shh expression, cells perceiving Shh signal are unlikely to require persistent Shh signaling for control of p53 activity, but rather only during a certain period of development. Shh is clearly not required to establish control of p53 activity since p53 can already be activated in the late gastrulation [40], which is much earlier than the 10somite stage. However, upon the onset of Shh expression, control of p53 pathway appears to become dependent on Shh signaling.

### Regulation of retinal cell proliferation by p53 in the *shh* ^−/−^ mutant

*p53* loss in the *shh* ^−/−^ mutant rescues two aspects of retinal proliferation: cell-cycle exit of retinal cell progenitors (Fig. 8) and the mitotic indices before and during neurogenesis (Fig. 7). Moreover, expression of the cyclin-dependent kinase inhibitor *p57kip2* is induced in the *p53* ^−/−^ *shh* ^−/−^ mutant retina (Fig. 8C) arguing for normal cell-cycle exit. A normal mitotic index in the *p53* ^−/−^ *shh* ^−/−^ mutant retina before neurogenesis (24 hpf) is likely to result from lack of p53-activated cell cycle inhibitory gene expression, while the rescue of cell-cycle exit and normal mitotic indices in the retina during neurogenesis may be due to both lack of cell-cycle inhibitor expression and more mitoses of differentiating cells upon cell-cycle exit. Previous studies of cell-cycle exit in the zebrafish retina [35], [36] showed that Hh signalling is essential for *p57kip2* expression in the retina. I support this conclusion and propose that Shh signalling regulates retinal expression of *p57kip2* by controlling activity of p53.

p53-mediated *Notch1* expression may be responsible for failure of *shh* ^−/−^ mutant embryos to express *p57kip2*. *Notch1* is a p53 target in human keratinocytes [58] and myeloid and lymphoid cells [59]. Moreover, bioinformatic analyses suggest that Gli2 can prevent p53 from activating *Notch1* gene expression, which could be a connection between p53, Hh and Notch pathways [60]. In zebrafish, *Notch1* homologs are also likely to be p53 targets since they contain optimal p53 binding sites (unpublished results). Notch signaling inhibits *p57kip2* expression during zebrafish neural tube development [61], during mouse pancreas development [62], in the adult mouse intestinal crypts [63] and during eye lense development in the mouse [64]. Whether p53 blocks retinal cell-cycle exit in the absence of *shh* by activating Notch signalling will undoubtedly be uncovered by future studies.

### Coordinate regulation of cell cycle, cell survival and differentiation in the retina

This study shows that p53 inhibits generation of amacrine and photoreceptor cells in the *shh* ^−/−^ mutant retina, raising a question whether p53 loss is essential only for their survival or also for differentiation. My data suggest that in the *shh* ^−/−^ mutant, *p53* loss is essential both for survival and cell-cycle exit of retinal progenitors, which must occur before cells can differentiate. By contrast, differentiation of ganglion cells, bipolar and Müller glia cells the *shh* ^−/−^ mutant retina is not affected by p53 suggesting that Shh signalling promotes retinal differentiation not only by controlling p53 activity but also by other p53-independent mechanisms.

In the retinal progenitor cells, differentiation decisions are coupled to regulation of cell cycle. This study has shown that *p53* affects both retinal cell cycle and differentiation phenotypes in the *shh* ^−/−^ mutant embryos implicating p53 pathway in coupling of cell cycle regulation and differentiation in the retina. Other studies identified some of the mechanisms coordinating cell cycle and differentiation decisions in the retina. Retinoblastoma 1 (Rb1) promotes retinal differentiation through inhibition of both E2f1 and E2f3a and its loss in the developing mouse retina leads to ectopic proliferation and elevated apoptosis rescued by E2f1 loss [65]. There is a functional analogy between Rb1-E2f and Shh-p53 interactions, Rb1 and Shh being required for proper cell cycle regulation, differentiation and control of E2f factors and p53, respectively. By contrast, in the postnatal mouse retina, Rb has been proposed to promote rod photoreceptor differentiation through activation of Nrl transcription factor [66]. CDK inhibitors (CDKI) are essential for cell-cycle exit of retinal progenitors, but are known regulate their differentiation through cell-cycle independent mechanisms, the importance of CDKI-mediated cell cycle inhibition still being unclear (reviewed in [67]). Studies by Ohnuma and colleagues showed that p27Xic1, a Xenopus CDK inhibitor, promotes generation of Müller glia at the expense of bipolar cells [68]. In this context, p27Xic1 interacts functionally with Notch/Delta signalling and proneural genes such as Xath5 to precisely regulate neurogenesis sequence [69], [70]. Consistent with the lack of amacrine cells in the *shh* ^−/−^ mutant, mouse p57Kip2 is involved in development of a subset of amacrine cells [71].

Regulation of cell death is another powerful mechanism known to shape organs. Several studies of zebrafish mutants addressed whether suppression of p53-mediated apoptosis affects retinal differentiation. In mutants of DNA polymerase delta 1 subunit (*pold1*), suppression of apoptosis in the retina by *p53* knockdown rescues eye size and morphology [72] and in the *primase1* mutant, *p53* knockdown led to the rescue of amacrine cell and photoreceptor differentiation [73]. By contrast, *p53* knockdown in *chromatin assembly factor 1b* mutant didn't improve retinal or cartilage differentiation [74], since this gene is required for both cell survival and differentiation of most cell types. Rescue of amacrine cells and photoreceptors by *p53* loss in the *shh* ^−/−^ mutant retina is similar to p53 knockdown results in *pold1* and *primase1* mutants, but Shh likely affects differentiation by regulating p53 activity and through other mechanisms, whereas mutations of *pold1* or *primase1* were proposed to induce genotoxic damage. Other studies identified context-dependence of p53 effects on differentiation, for example, p53 promotes differentiation in mouse embryonic stem cells after DNA damage by decreasing Nanog expression [75] and reduces spontaneous differentiation of human stem cells [76].

### Conclusions

These results identify p53 pathway as the essential mediator of the elevated apoptosis in the developing nervous system and retina in the absence of Shh in zebrafish. I also find that Shh signalling promotes cell survival in this context by inhibiting p53 pathway. In the retina, Shh controls p53 to regulate both cell survival and proliferation, since in the *shh* mutant loss of *p53* suppresses elevated apoptosis, leads to normal mitotic indices in the retina and rescues cell-cycle exit. Control of p53 by Shh is also essential for differentiation of some amacrine cells and photoreceptors.

## Materials and Methods

### Fish stocks, maintenance, identification and care

Wild-type *WIK*, *sonic-you* ^t4^ [77] and *p53^zdf1^* [44] heterozygous fish were used. Fish were maintained according to standard protocols. Embryos were grown in E3 embryo medium at 28°C with or without the addition of 0.003% 1-phenyl-2-thiourea (Sigma) to inhibit pigmentation. Staging was performed according to hours post-fertilization (hpf) [78]. Identification of *p53* genotype was performed according to a published protocol [44]. Fin clips were obtained by anesthesizing adult fish in 0.02% Tricaine and then cutting small pieces of the caudal fin. All DNA samples for identification were prepared as described previously [79]. *sonic-you* ^t4^ mutants were identified by their curled body shape and U-shaped somites.

### Morpholino and mRNA microinjection procedures

p53 morpholino (GCGCCATTGCTTTGCAAGAATTG, [42])and four-mismatch control p53 morpholino (GCA CCA TCG CTT GGC AAG CAT TG) [72] were purchased from Gene Tools and injected into 1-cell stage at 0.5 mM. To make RNA for over-expression experiments, required pCS2+ vectors were linearised and transcribed using mMessage mMachine SP6 (Ambion, cat # AM1340). The following plasmids were used to prepare RNA for microinjections: pCS2+dnPKA-GFP [51], pCS2+EGFP, pCS2+EGFP-bcl2 [54], pCS2+FA Transposase (Tol2kit).

### Generation of the PG13p21::nlsEGFP p53 reporter line

Human CDKN1A/p21 2.4 kb promoter was amplified from RPCI-11 Human BAC clone (Imagenes, RPCIB753F15397Q) using the following primers:

HindIII_p21prom_for AAGCTTGGGCACTCTTGTCCCCCAGG

BamHI_p21prom_rev GGATCCCTGACTTCGGCAGCTGCTCA

The resulting PCR product was TOPO-cloned into pCR2.1 (Invitrogen) and then subcloned into the p5E-MCS vector using HindIII and BamHI restriction sites to make p5E-p21 vector. PG13 enhancer (13 copies of an optimal p53 binding site CCTGCCTGGACTTGCCTGG) [46] was ordered from GENEART AG (custom product). The synthetic PG13 fragment contained KpnI and HindIII restriction sites for directional cloning into p5E-p21. Insertion of PG13 into p5E-p21 resulted into the final 5′-entry vector p5E-PG13p21. To generate pDestCG2-PG13p21::nlsEGFP-polyA, p5E-PG13p21 was recombined with pME-nlsEGFP, p3E-polyA and pDestCG2 vectors (Tol2kit, [47]). The construct was then injected into wild-type embryos, which were raised to adulthood and identified as transgenic founders.

### *In situ* hybridisation and probe synthesis

RNA in situ hybridisations were performed according to Jowett (2001) [80]. Probes were made using Roche DIG RNA Labeling Kit (Cat # 11175025910). The fragments of *p53*, *puma*, *p21*, *bcl2*, *mcl1a*, *mcl1b* were cloned into pCR4 vector (Invitrogen) using the primers listed in Table 1. Plasmids containing *cyclinG1* cDNA (pCMV-SPORT6.1-cyclinG1 (Imagenes)), *mdm2* cDNA (pME-18S-FL3 – mdm2 (Imagenes)), *bax1* cDNA (pME-18S-FL3 – bax1 (Imagenes)) and *bcl2l* (pDNR-LIB-bcl2l (Imagenes)) were used for making templates for *in vitro* transcription using primers listed in Table 1. Probes for *p57* and *patched1* were made using previously published plasmids [35], [81].

**Table 1:** Primers used to amplify cDNA fragments.

Primer_name	Sequence	Template for PCR
mcl1a_for	tatggctttgagtttggattttagg	cDNA
mcl1a_rev	gcattgatagccattggc	cDNA
mcl1b_for	gagaagaagaaaaaatgtagagttg	cDNA
mcl1b_rev	agcacttcgaaaaggaaaa	cDNA
bcl2l_for	tggataaccgtattaccgcc	pDNR-LIB-bcl2l (ZFIN)
T3_bcl2l_rev	cgcgcaattaaccctcactaaagcactagtcataccaggatc	pDNR-LIB-bcl2l (ZFIN)
bcl2_for	gcgcgtttctatcgtgattt	cDNA
bcl2_rev	agcatgtgtgcacgtgtttt	cDNA
bax1_for	tgtacggaagtgttacttctgctc	pME18S-FL3-bax1(ZFIN)
T3_bax1_rev	ggatccattaaccctcactaaagggaaggccgcgacctgcagctc	pME18S-FL3-bax1(ZFIN)
p53_for	gaatccccaaaactccacgc	cDNA
p53_rev	ccaaaaagagcaaaactccc	cDNA
pME_for	attaaccctcactaaaggctgctcctcagtggatgttgcctttac	pME18S-FL3-mdm2 (Imagenes)
T7_pME_rev	taatacgactcactataggcaggttcagggggaggtgtgg	pME18S-FL3-mdm2 (Imagenes)
puma_for	aacaccgctttatatcccct	cDNA
puma_rev	tgtgttcatctgaggccgtg	cDNA
p21_for	tcaggtgttcctcagctcct	cDNA
p21_rev	cggaataaacggtgtcgtct	cDNA

### BrdU labelling

Embryos were anesthesized with 0.02% Tricaine and injected with a small drop (5–10 nl) of 10 mM 5-Bromodeoxyuridine (BrdU) into the yolk sac. After injection, embryos were allowed to develop for a required period of time and fixed in PFA for analysis. BrdU-labeled embryo were then cryosectioned and the sections were dried, washed in PBST (PBS + 0.1% Tween20 (Sigma, cat# P9416)), incubated in 2N HCl for 10 minutes at 23°C and then transferred to 37°C for another 30 minutes and washed again in PBST. Anti-BrdU staining was done as described in the next section of Materials and Methods, the sections finally being stained with 20 µg/ml of DAPI. Quantification of BrdU-positive nuclei was done using “Colocalization Threshold” analysis of BrdU and DAPI signals and “Cell Counter” plugins of Fiji software (http://pacific.mpi-cbg.de) as described below.

### Immunohistochemical methods

Antibody stainings were done on cryosections as described before [35]. The following antibodies were used: anti-zn5, anti-zpr1, anti-zpr3 at 1∶100 dilution (Developmental Studies Hybridoma Bank), anti-parvalbumine – Chemicon (MAB1572) at 1∶200 dilution, anti-GAD67 – Chemicon (AB5862P) at 1∶100 dilution, anti-glutamine synthetase – BD Biosciences (cat# 610517) at 1∶500 dilution, anti-PKCα – Santa Cruz Biotechnology (sc-208) at 1∶100 dilution, anti-HuC – Molecular Probes (A-21271), anti-GFP – Molecular Probes, Invitrogen (A11122) at 1∶200 dilution, anti-BrdU – Sigma (cat# B8434) at 1∶100 dilution, anti-pH3-S10 – Abcam (ab5176-100) at 1∶400 dilution, anti-activeCaspase3 – BD Pharmingen (cat# 557035) at 1∶200 dilution. Secondary anti-mouse Alexa Fluor 488 antibody (1∶400) (Invitrogen, cat# A11001), anti-rabbit Alexa Fluor 488 (1∶400) (Invitrogen, cat# A-11008) and anti-rabbit Alexa Fluor 594 (1∶400) (Invitrogen, cat# A-11012) were diluted in blocking solution and incubated on the sections for 2 hours. To label actin structures, Alexa Fluor 568 labeled phalloidin (Invitrogen, A12380) was diluted at 1∶400 in blocking buffer and added to secondary antibody solutions. Images were taken using Leica SP2 confocal microscope and processed using Adobe Creative Suite CS4.

### Co-localisation analysis

Co-localisation was analysed using “Colocalisation Threshold” plugin of Fiji software. The plugin takes two images and outputs several statistics including percent of co-localised pixels relative to total pixels in each of the co-localising channels and an image, where pixels present in both images are white.

### Apoptosis assays

ApopTag staining was performed using Chemicon ApopTag Peroxidase in situ apoptosis detection kit (Cat # S7100) as described before [74]. TUNEL staining on cryosections was performed as described before [35] using TUNEL-TMR-Red kit (Roche, cat # 1966006001).

### qPCR and semi-quantitative PCR assays

RNA from whole embryos or embryo heads was purified using RNeasy kit (Qiagen, cat# 74104) and cDNA samples were prepared using Quantitect Reverse Transcription Kit (Qiagen, cat# 205311) and used for quantitative real-time PCR after 20-fold dilution. SYBR® Green PCR Master Mix (Applied Biosystems, cat# 4309155) was used to prepare qPCR reactions, which were run on the ABI7500 qPCR machine. Primers used for qPCR assays and appropriate references are listed in Table 2. Semi-quantitative PCR for full-length *p53*, its Δ113 isoform and *b-actin* transcripts was performed essentially as described before [38].

**Table 2:** Primers for qPCR assays.

Primer	Sequence	Source
p53q_for	cccatcctcacaatcatcact	This study
p53q_rev	cacgcacctcaaaagacctc	This study
p21_qfor	agctgcattcgtctcgtagc	[72]
p21_qrev	tgagaacttactggcagcttca	[72]
mdm2q_for	gatgcaggtgcagataaagatg	This study
mdm2q_rev	ccttgctcatgatatatttccctaa	This study
cycG1-qfor	catctctaaaagaggctctagatgg	This study
cycG1-qrev	cacacaaaccaggtctccag	This study
bax1_qfor	gcaagttcaactggggaaga	This study
bax1_qrev	gtcaggaaccctggttgaaa	This study
gapdh_qfor	tgcgttcgtctctgtagatgt	[40]
gapdh_qrev	gcctgtggagtgacactga	[40]

### Counting procedure

TUNEL-positive, DAPI-stained nuclei and pH 3-labelled cells were counted manually using the Cell Counter plugin of ImageJ, which allows labelling already counted objects and automatically records their number.

### Image quantification

Image quantification in Figs. 2A-B and Fig.9 was done using Fiji software. To quantify the area of particular signal in an image, raw RGB images were split into channels, and single channel images were processed to remove irrelevant parts of the image (select and Edit Clear Outside). The processed images were thresholded, the threshold being the same for all images in an experiment and manually chosen for optimal segmentation of the signal. Thresholded images were then segmented using the Analyze Particles plugin (Analyze Analyze Particles), which outputs the area of the segmented signal.

### Statistical analyses

The differences of cell proportions and counts were statistically evaluated using Student's t-test in Microsoft Excel 2007. qPCR data were initially processed using the SDS1.2 software from Applied Biosystems to obtain Ct values, that is the amplification cycle number at which a reaction reaches a certain threshold of expression. Relative gene expression was calculated using the ΔΔCt method ([82]) assuming equal amplification efficiencies. Ct values of all samples were normalised by reference gene expression and by average wild-type gene expression before being converted to fold expression differences. Student's t-test was then applied to fold expression differences (p<0.05 was set as a significance level). Statistical significance of embryo proportions in the Hedgehog signalling activation experiment (Fig. 6) was determined using Fisher's exact test using fisher.test function from R language environment (R version 2.9.0).

## Supporting Information

Figure S1 Expression of anti-apoptotic Bcl2 family genes in shh−/− mutant versus wild-type zebrafish embryos at 24 hpf. In situ whole-mount analysis of bcl2, bcl2l, mcl1a and mcl1b expression in shh−/− mutant and wild-type embryos at 24 hpf.
(3.22 MB TIF)

Figure S2 Onset of increased p53 expression and apoptosis in shh−/− mutant occurs during somitogenesis. p53 in situ was used to identify time points with activated p53 protein (A, B, E, F, I, J, M, N, R, S, V, W). ApopTag staining was used as an assay for apoptosis (C, D, G, H, K, L, P, Q, T, U, X, Y). The following stages were analysed 1somite (A–D), 6somite (E–H), 8somite (I–L), 10somite (M–Q), 12somite (R–U), 14somite (V–Y). At early stages it was not possible to identify the embryos (wild-type or shh−/−). From 10somite stage, shh−/− mutant embryos could be identified based on their p53 expression and ApopTag staining because they made one quarter of the batch. The stainings were repeated two times and 40 embryos were analysed for each staining.
(5.97 MB TIF)

Figure S3 Normal differentiation of retinal cell types in p53−/−mutant. Antibody staining of retinal cryosections from p53−/− mutant embryos at 72 hpf. Anterior side of the sections is to the top of the images. Phalloidin-Alexa568 was used to label actin structures. The images show normal differentiation of ganglion cells (zn-5), amacrine cells (GAD67 and parvalbumin), Müller glia (glutamine synthetase (GS)), bipolar cells (Protein kinase C α (PKCa)) and photoreceptors (zpr1 and zpr3).
(3.13 MB TIF)
