# Supplementary figures and images for: In the Absence of Sonic Hedgehog, p53 Induces Apoptosis and Inhibits Retinal Cell Proliferation, Cell-Cycle Exit and Differentiation in Zebrafish
Source: PLoS One. 2010 Oct 21;5(10):e13549. doi: 10.1371/journal.pone.0013549 (PMC2958845; doi:10.1371/journal.pone.0013549)

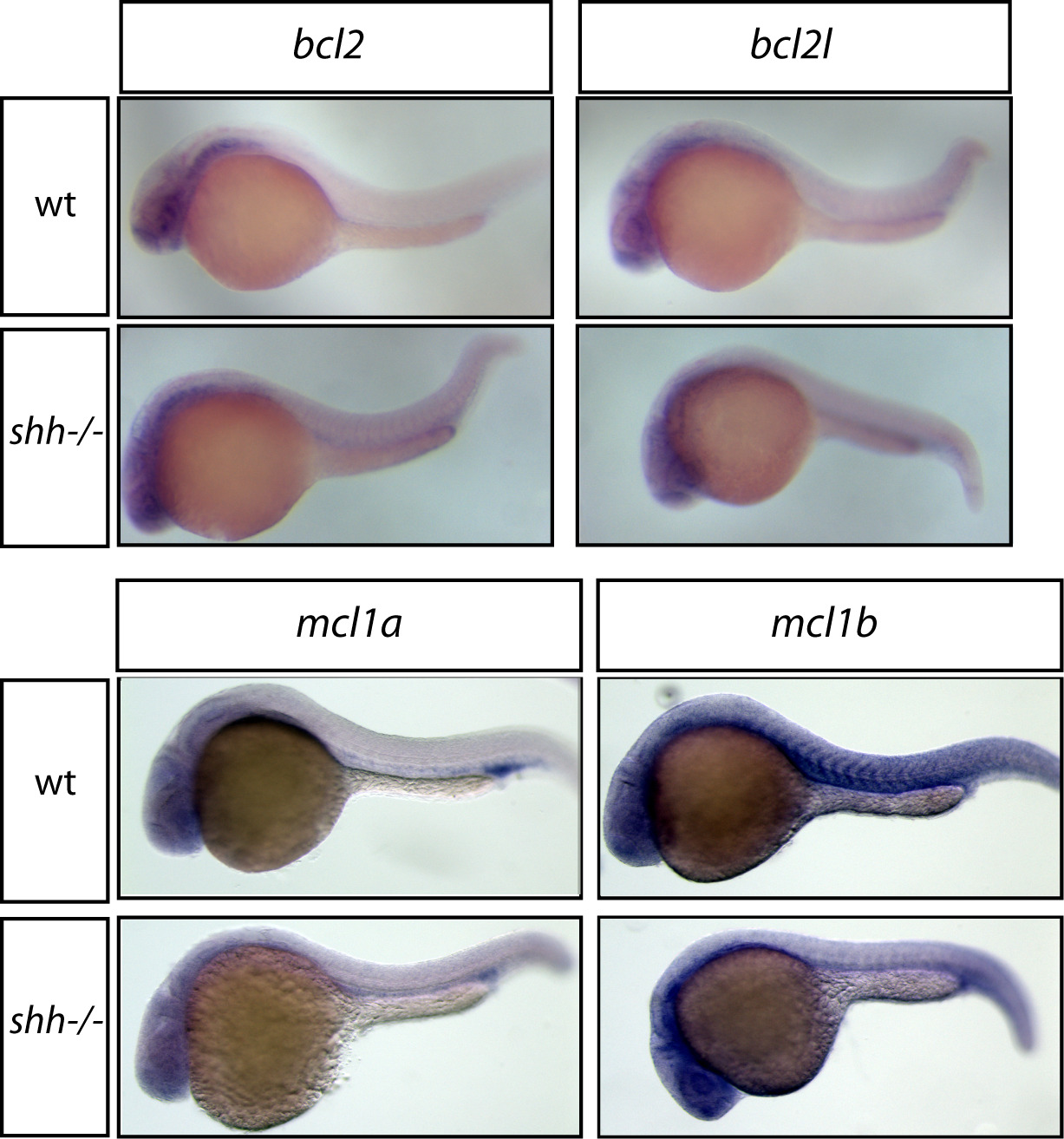

Supplement: Figure S1 — Expression of anti-apoptotic Bcl2 family genes in shh−/− mutant versus wild-type zebrafish embryos at 24 hpf. In situ whole-mount analysis of bcl2, bcl2l, mcl1a and mcl1b expression in shh−/− mutant and wild-type embryos at 24 hpf. (3.22 MB TIF) [file pone.0013549.s001.tif]

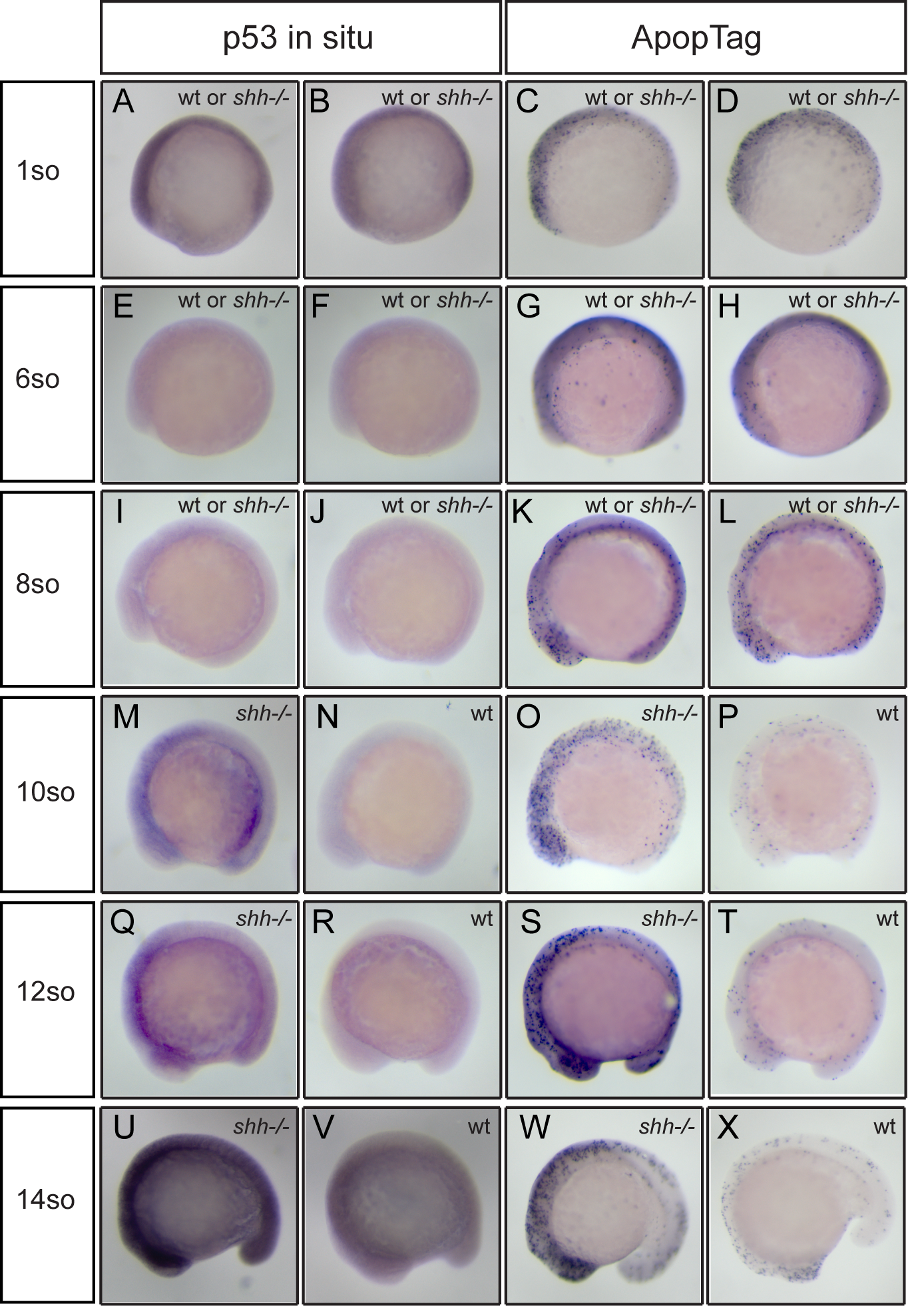

Supplement: Figure S2 — Onset of increased p53 expression and apoptosis in shh−/− mutant occurs during somitogenesis. p53 in situ was used to identify time points with activated p53 protein (A, B, E, F, I, J, M, N, R, S, V, W). ApopTag staining was used as an assay for apoptosis (C, D, G, H, K, L, P, Q, T, U, X, Y). The following stages were analysed 1somite (A–D), 6somite (E–H), 8somite (I–L), 10somite (M–Q), 12somite (R–U), 14somite (V–Y). At early stages it was not possible to identify the embryos (wild-type or shh−/−). From 10somite stage, shh−/− mutant embryos could be identified based on their p53 expression and ApopTag staining because they made one quarter of the batch. The stainings were repeated two times and 40 embryos were analysed for each staining. (5.97 MB TIF) [file pone.0013549.s002.tif]

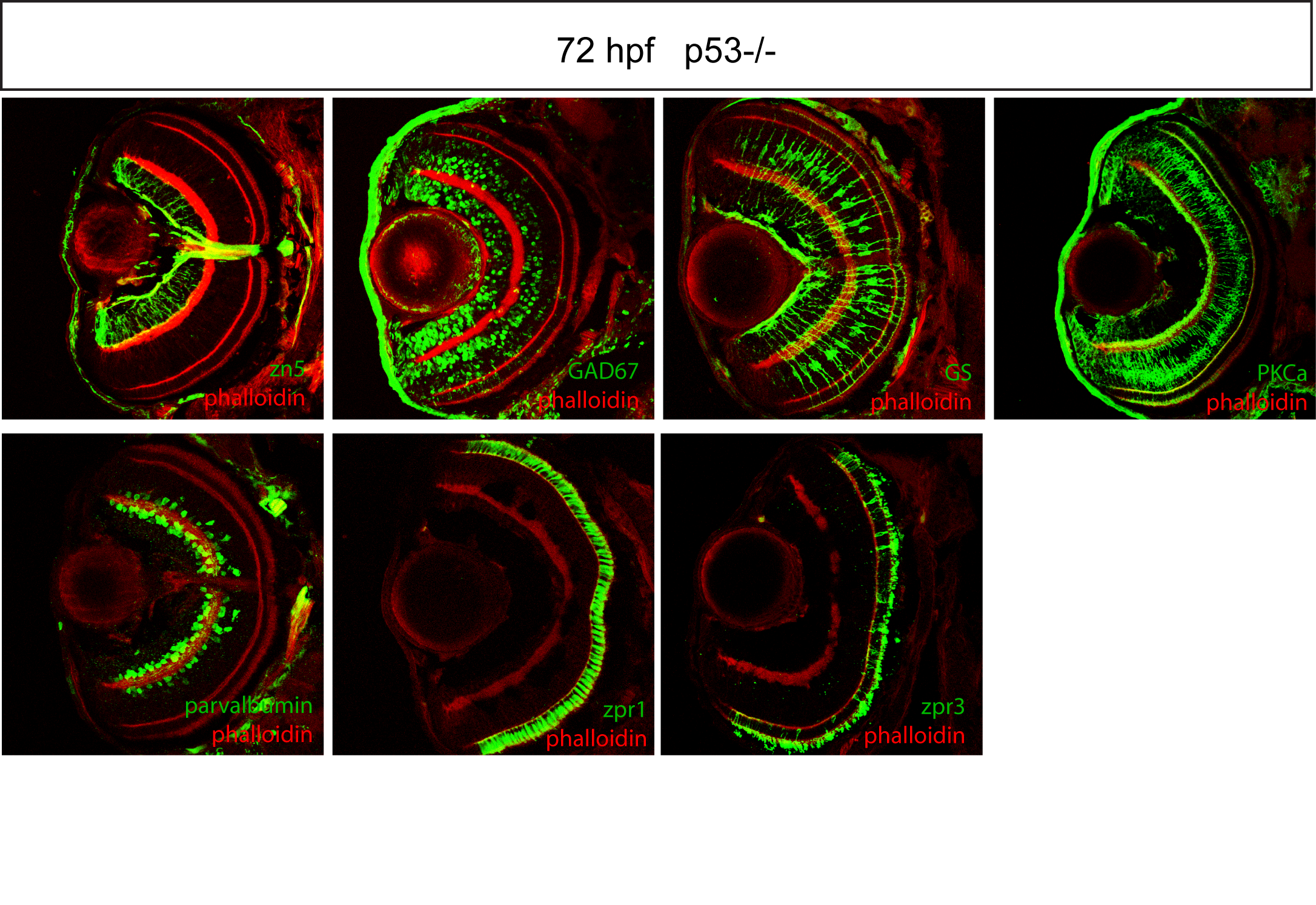

Supplement: Figure S3 — Normal differentiation of retinal cell types in p53−/−mutant. Antibody staining of retinal cryosections from p53−/− mutant embryos at 72 hpf. Anterior side of the sections is to the top of the images. Phalloidin-Alexa568 was used to label actin structures. The images show normal differentiation of ganglion cells (zn-5), amacrine cells (GAD67 and parvalbumin), Müller glia (glutamine synthetase (GS)), bipolar cells (Protein kinase C α (PKCa)) and photoreceptors (zpr1 and zpr3). (3.13 MB TIF) [file pone.0013549.s003.tif]
